# ER stress upregulates S100A11 in steatohepatitis models via epigenetic modifications within the lipotoxicity-influenced enhancer

**DOI:** 10.1172/JCI191074

**Published:** 2025-09-30

**Authors:** P. Vineeth Daniel, Hanna L. Erickson, Daheui Choi, Feda H. Hamdan, Yasuhiko Nakao, Gyanendra Puri, Takahito Nishihara, Yeriel Yoon, Amy S. Mauer, Debanjali Dasgupta, Jill Thompson, Alexander Revzin, Harmeet Malhi

**Affiliations:** 1 Division of Gastroenterology and Hepatology,; 2 Department of Physiology and Biomedical Engineering, and; 3 Department of Molecular Medicine, Mayo Clinic, Rochester, Minnesota, USA.

**Keywords:** Hepatology, Inflammation, Metabolism, Epigenetics

## Abstract

Metabolic dysfunction–associated steatohepatitis (MASH) is a progressive liver disease characterized by complex interactions between lipotoxicity, ER stress responses, and immune-mediated inflammation. We identified enrichment of the proinflammatory alarmin S100 calcium-binding protein A11 (S100A11) on extracellular vesicles stimulated by palmitate-induced lipotoxic ER stress with concomitant upregulation of hepatocellular S100A11 abundance in an IRE1A-XBP1s–dependent manner. We next investigated the epigenetic mechanisms that regulate this stress response. Publicly available human liver ChIP-Seq GEO datasets demonstrated a region of histone H3 lysine 27 (H3K27) acetylation upstream of the *S100A11* promoter. H3K27 acetylation ChIP-qPCR demonstrated a positive correlation between lipotoxic ER stress and H3K27 acetylation of the region, which we termed the lipotoxicity-influenced enhancer (LIE) domain. CRISPR-mediated repression of the LIE domain reduced palmitate-induced H3K27 acetylation and corresponding S100A11 upregulation in Huh7 cells and immortalized mouse hepatocytes. Silencing of the murine LIE in 2 independent steatohepatitis models demonstrated reduced *S100a11* upregulation and attenuated liver injury. We confirmed H3K27 acetylation and XBP1s occupancy at the LIE domain in human MASH liver samples and an increase in hepatocyte-derived S100A11-enriched extracellular vesicles in MASH patient plasma. Our studies demonstrate a LIE domain that mediates hepatic S100A11 upregulation. This pathway may be a potential therapeutic target in MASH.

## Abstract 2

We discovered how a key protein S100A11 is regulated and drives progression of fatty liver disease, revealing a new target for future treatments.

## Introduction

Metabolic dysfunction–associated steatohepatitis (MASH) is the most prevalent chronic liver disease worldwide. MASH involves the accumulation of lipids, termed steatosis, along with inflammation and liver damage, known as lipotoxicity. Damaged liver cells release soluble mediators like damage-associated molecular patterns (DAMPs) or alarmins that stimulate a localized chronic sterile inflammatory response (1, 2). Alarmin proteins execute dual functions, maintaining cellular homeostasis while also activating inflammatory mechanisms in response to tissue damage signals. High-mobility group box 1, IL-1α, IL-33, and the S100 family of proteins are well-reported alarmin proteins (3). Both parenchymal and non-parenchymal liver cells sense alarmins via pattern recognition receptors, leading to the infiltration of proinflammatory systemic immune cells into the liver. In brief, hepatic lipotoxicity serves as a critical metabolic stressor that stimulates inflammation in the liver via the release of alarmin proteins (4).

ER stress is a well-established feature of lipotoxicity in MASH (5). Lipotoxic ER stress–induced cell dysfunction and lipoapoptosis are associated with sterile inflammation in the liver (6). Recently, we have reported the release of inflammatory DAMP-containing extracellular vesicles (EVs) from lipotoxic hepatocytes (7, 8). EVs are cell-specific membranous nanovesicles that feature differential cargo and composition under stress conditions, imparting stress- and tissue-specific characteristics (9). In this context, our laboratory has previously demonstrated that lipotoxic ER stress mediates EV release from hepatocytes in an inositol-requiring enzyme 1α–dependent (IRE1A-dependent) manner (10). Additionally, we have reported that ceramide-dependent lipotoxic EVs are enriched with DAMPs (11). Given that MASH is inherently lipotoxic and exhibits elevated intrahepatic inflammatory signaling, we anticipated that lipotoxic EV cargos may be a pathogenically relevant pool of candidate proteins in MASH.

The susceptibility and variability of MASH progression and response to therapeutic agents can vary substantially across individuals. The unpredictable rate of MASH progression suggests that genetic or epigenetic factors may explain individual variability. While GWAS and population genetics highlight genetic variants that could promote the onset or progression of MASH, there is limited knowledge of the epigenetic determinants of disease progression, which have emerged as a key mechanism that could explain the impact of dynamic and non-genetic factors on MASH progression (12, 13). Epigenetic mechanisms can regulate the transcriptomic perturbations elicited by lipotoxicity (14). A few studies have demonstrated an association of epigenetic changes with MASH, identifying high-calorie diet as having remodeled the DNA methylome, subsequently leading to hepatic steatosis (15, 16). However, the molecular mechanisms of epigenetic regulators like histone code and DNA methylation, microRNAs, and long non-coding RNAs in MASH pathogenesis and progression are not well understood (14, 17). Since epigenetic regulation demonstrates cell and stimulus specificity (18), similarly to the ER stress response, a crosstalk between lipotoxic ER stress and epigenetic mechanisms may determine critical characteristics of MASH.

Integrating hepatocyte EV alarmins with individual variability, we asked whether a lipotoxic ER stress–dependent epigenetically regulated alarmin may modulate liver inflammation in MASH. Here, we report that lipotoxic ER stress upregulated the expression of S100 calcium-binding protein A11 (S100A11), within both the cytosolic and EV pool, in a manner dependent on IRE1A-spliced X-box–binding protein 1 (XBP1s). Additionally, we observed that lipotoxic ER stress stimulates an epigenetic response by histone H3 lysine 27 acetylation (H3K27ac) of an enhancer element upstream of the *S100A11* promoter. CRISPR interference–mediated (CRISPRi-mediated) repression of this transcriptional complex lowered hepatic *S100a11* expression and mitigated steatohepatitis in murine MASH models. We additionally confirmed that this epigenetic regulation is observed in human MASH livers. Overall, our study identifies S100A11 as a hepatocyte lipotoxicity–specific druggable target, regulated by a functional lipotoxicity-influenced enhancer (LIE) DNA element, which may explain individual variability in MASH.

## Results

### S100A11 is enriched in hepatic lipotoxic EVs.

**Figure 1:**
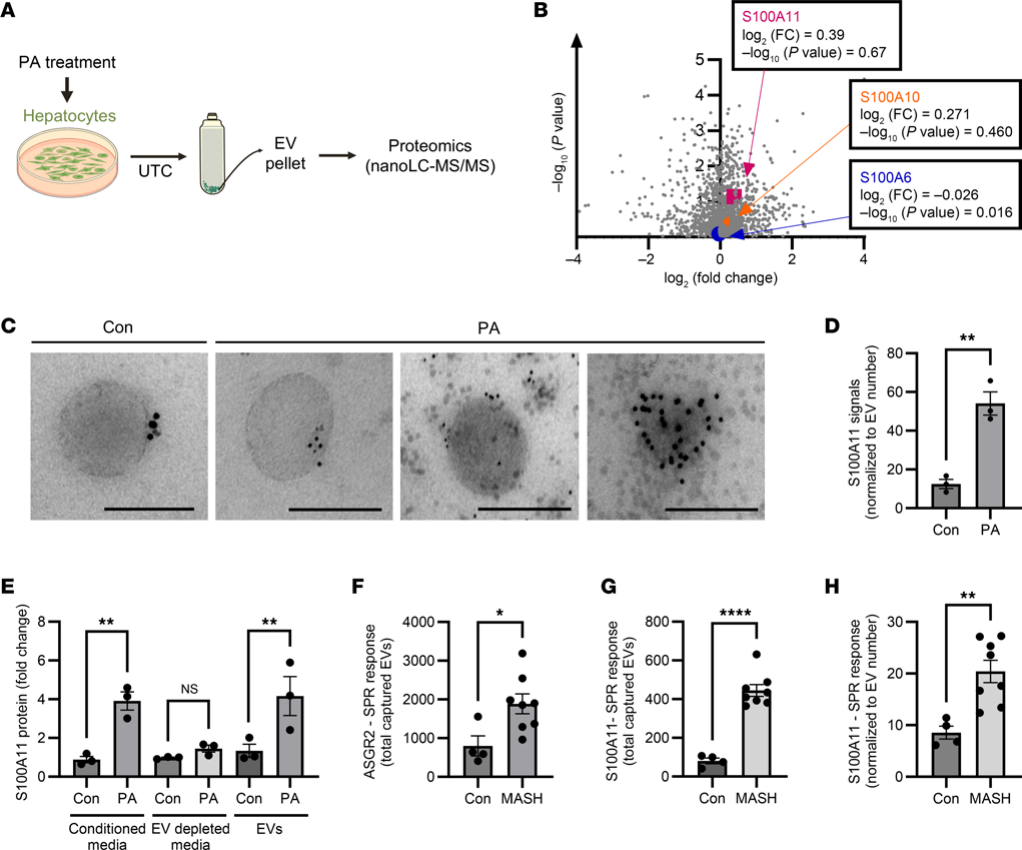
S100A11 is enriched in hepatic EVs. (**A**) Schematic representation of untargeted EV proteomics protocol following ultracentrifugation (UTC) and nano–liquid chromatography–tandem mass spectrometry (nanoLC-MS/MS). (**B**) Volcano plot of EV proteomics performed using EVs collected from WT-IMH cells treated with 400 μM palmitate (PA) for 16 hours compared with vehicle-treated controls (*n* = 3 per group). S100A11 (pink square), S100A10 (orange triangle), and S100A6 (blue circle) are indicated as differentially enriched. Student’s unpaired 2-tailed *t* tests were used to compare 2 groups. (**C**) Immunogold labeling of S100A11 (black dots) on EVs from supernatants of PMHs treated with 400 μM PA or vehicle for 16 hours. Representative data are shown from *n* = 3 experimental replicates. Scale bars: 200 nm. (**D**) SPR-based quantification of S100A11 signals in EVs isolated from PMHs treated with 400 μM PA for 16 hours compared with vehicle-treated controls (*n* = 3 per group). SPR response is expressed in resonance units (RU). Student’s unpaired 2-tailed *t* test was used to compare 2 groups. (**E**) ELISA-based quantification of S100A11 protein in conditioned medium, EV-depleted medium, and EVs from Huh7 cells treated with 600 μM PA for 20 hours compared with vehicle-treated controls (*n* = 3 per group). One-way ANOVA with Šidák’s multiple-comparison test was used to compare groups. (**F**) SPR-based quantitative evaluation of ASGR2-expressing hepatic EVs isolated from plasma samples from both MASH (*n* = 8) and control (*n* = 4) patients. SPR response is expressed in RU. Student’s unpaired 2-tailed *t* test was used to compare 2 groups. (**G** and **H**) SPR-based quantification of S100A11 abundance on ASGR2-captured hepatic EVs from MASH (*n* = 8) and control (*n* = 4) plasma samples, expressed without (**G**) and with (**H**) EV number normalization. Student’s unpaired 2-tailed *t* test was used to compare 2 groups. *P < 0.05, **P < 0.01, ****P < 0.0001.

To investigate DAMPs enriched in secreted EVs of lipotoxic hepatocytes, we used EV proteomics as previously published by us (11). We treated an immortalized, non-transformed, wild-type hepatocyte cell line, termed immortalized mouse hepatocytes (WT-IMH) (11, 19, 20), with palmitate or vehicle and isolated EVs by differential ultracentrifugation of cell culture supernatants followed by untargeted proteomics (Figure 1A). Bioinformatic analysis of the reads yielded 1,660 proteins to be differentially expressed, as shown in the volcano plot (Figure 1B). Focusing on DAMPs (21), we observed 44 proteins to be differentially expressed, among which S100A11 was upregulated, supporting our previous finding of S100A11 abundance as a ceramide-dependent EV cargo (11). Among the S100 family of proteins, S100A6 and S100A10 were also differentially expressed, albeit to a lower fold than S100A11 (Figure 1B) (3, 22).

We next evaluated S100A11 expression on EVs using a combination of qualitative and quantitative methods, including electron microscopy (EM), surface plasmon resonance (SPR), Western blotting, and ELISA. Using immunogold EM and antibodies against S100A11, we observed an increase in S100A11 on the surface of palmitate-stimulated primary mouse hepatocyte–derived (PMH-derived) EVs (Figure 1C) compared with the vehicle group (23). To quantify S100A11 abundance on EVs, we used an SPR- and antibody-based detection method. In this approach (Supplemental Figure 1A; supplemental material available online with this article; https://doi.org/10.1172/JCI191074DS1), 2 × 10^9^ EVs collected from palmitate- or vehicle-treated PMHs were infused into the SPR instrument, wherein EVs were captured on an SPR chip functionalized with an anti–asialoglycoprotein receptor 1 (anti-ASGR1) antibody, a hepatocyte-specific marker (24). Next, the S100A11 signals on the captured EVs were quantified using SPR and anti-S100A11 antibody. We observed that EVs isolated from the palmitate-stimulated PMHs had enrichment of S100A11 signals compared with EVs isolated from the vehicle-treated PMHs (Figure 1D). To confirm EV enrichment of S100A11 in a human cell line, Huh7 cells were treated with palmitate. Western blotting of the EV lysates demonstrated increased S100A11 expression in EVs isolated from palmitate-treated Huh7 cells (Supplemental Figure 1B). Furthermore, we measured S100A11 protein by ELISA in the Huh7-conditioned cell culture supernatants containing EVs and following EV depletion. Palmitate-treated Huh7 cell culture supernatants displayed higher S100A11 protein levels (Figure 1E). In cell culture supernatants depleted of EVs, there was no statistically significant difference in S100A11 levels between control and palmitate supernatants (Figure 1E). Yet, EVs isolated from these samples demonstrated an increase in S100A11 in palmitate-treated Huh7 EVs, confirming the preferential secretion of S100A11 in EVs (Figure 1E). Thus, palmitate-induced lipotoxicity leads to the release of S100A11-enriched EVs.

Identification of S100A11 as an EV-bound DAMP secreted from lipotoxic hepatocytes made us question whether MASH patient plasma samples also exhibited increased S100A11 on circulating hepatocyte-derived EVs. To answer this question, we measured hepatic S100A11 in EVs from plasma samples of MASH patients using SPR technique. EVs were isolated using differential ultracentrifugation. There was an increase in EVs in MASH plasma samples compared with the control plasma samples when quantified by Nanoparticle Tracking Analysis (Malvern Panalytical), consistent with our earlier data (Supplemental Figure 1C) (10). Asialoglycoprotein receptor 2–expressing (ASGR2-expressing) EVs, which are hepatocyte derived (24, 25), were higher in MASH plasma samples compared with control samples (Figure 1F). Normalization of SPR signals based on EV numbers demonstrated equivalent EV capture among the 2 groups (Supplemental Figure 1D). Taken together, these data confirm that MASH plasma samples have increased numbers of hepatic EVs compared with the control. Labeling of captured EVs with an anti-S100A11 antibody revealed higher S100A11 SPR signal for MASH samples compared with control samples with and without EV number normalization (Figure 1, G and H). Further labeling of the ASGR2-captured, S100A11-labeled EVs with anti-CYP2E1 antibody (a second independent hepatocyte-specific marker) confirmed MASH samples to have increased S100A11 on dual-labeled (ASGR2- and CYP2E1-labeled) hepatic EVs compared with the controls (Supplemental Figure 1E). Thus, these data demonstrate that hepatocyte-derived S100A11 is a highly upregulated lipotoxic EV cargo in MASH.

### IRE1A regulates lipotoxic ER stress–mediated S100A11 upregulation.

**Figure 2:**
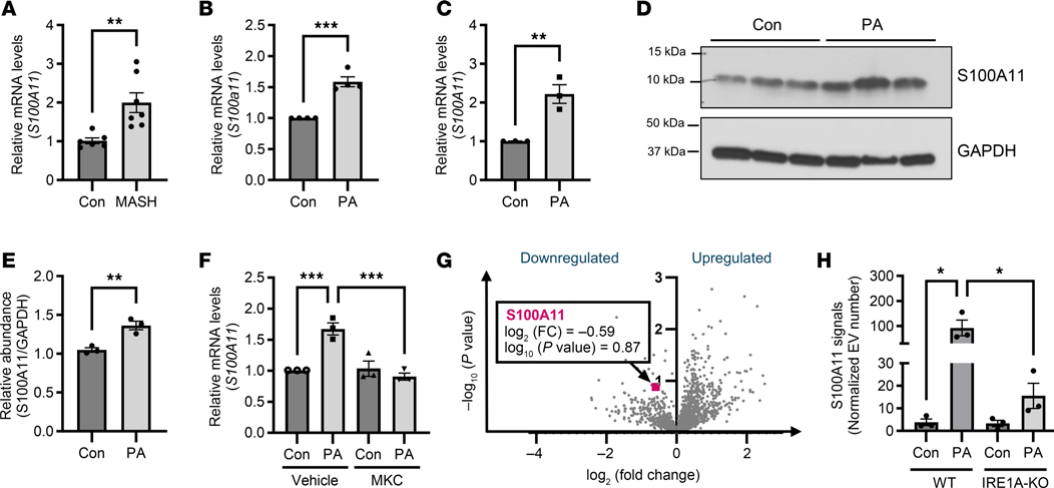
IRE1A regulates lipotoxic ER stress–mediated S100A11 upregulation. (**A**) Expression of *S100A11* transcript in liver from human MASH patients (*n* = 7) and normal controls (*n* = 6). Relative fold change was calculated using 18S as the loading control. Student’s unpaired 2-tailed *t* test was used to compare 2 groups. (**B**) Expression of *S100a11* transcript in PMHs treated with 400 μM palmitate (PA) for 16 hours compared with vehicle-treated controls (*n* = 4 per group). Student’s unpaired 2-tailed *t* test was used to compare 2 groups. (**C**) Expression of *S100A11* transcript in Huh7 cells treated with 600 μM PA for 16 hours compared with vehicle-treated controls (*n* = 3 per group). Student’s unpaired 2-tailed *t* test was used to compare 2 groups. (**D**) Western blot analysis of S100A11 expression in Huh7 cells treated with 600 μM PA for 16 hours compared with vehicle-treated controls (*n* = 3 per group). (**E**) Western blot quantification by densitometry. Student’s unpaired 2-tailed *t* test was used to compare 2 groups. (**F**) Expression of *S100A11* transcript in Huh7 cells treated with 600 μM PA for 16 hours in the presence and absence of IRE1A inhibition with 10 μM MKC8866 (MKC) (*n* = 3 per group). Two-way ANOVA with Šidák’s multiple-comparison test was used to compare 2 groups with 2 treatments. (**G**) Volcano plot of EV proteomics using EVs collected from IRE1A-KO-IMH cells treated with 400 μM PA for 20 hours compared with WT-IMH PA–treated controls (*n* = 3 per group). S100A11 indicated by pink square. Student’s unpaired 2-tailed *t* test was used to compare 2 groups. (**H**) SPR-based quantification of S100A11 signals in EVs isolated from WT-IMH and IRE1A-KO-IMH cells treated with 400 μM PA for 20 hours compared with vehicle-treated controls (*n* = 3 per group). SPR response is expressed in resonance units (RU). One-way ANOVA with Šidák’s multiple comparisons was used to compare groups. *P < 0.05, **P < 0.01, ***P < 0.001.

Increased cellular expression of EV cargo proteins is one mechanism that can lead to their enrichment in EVs. Therefore, we asked whether lipotoxic conditions stimulated the upregulation of S100A11, leading to its subsequent release on hepatocyte-derived EVs. Assessment of the human MASH livers demonstrated upregulation of *S100A11* transcripts in MASH livers compared with control livers (Figure 2A). Palmitate-treated PMHs also exhibited upregulation of *S100a11* transcripts compared with vehicle-treated controls (Figure 2B), consistent with previous studies in human MASH and free fatty acid–treated hepatocyte cell lines (26, 27). To elucidate the hepatocellular signaling mechanisms that mediate transcriptional upregulation of S100A11 in lipotoxic conditions, we next used Huh7 cells. Similarly to human MASH and palmitate-treated PMH cells, we noted that palmitate-treated Huh7 cells had enhanced *S100A11* mRNA expression, in comparison with the control (Figure 2C). Palmitate-treated Huh7 also had increased cytosolic S100A11 protein levels demonstrated by Western blotting and densitometry (Figure 2, D and E).

Increased levels of circulating saturated free fatty acids in metabolic diseases like MASH are reported to activate the unfolded protein response (UPR) sensors by lipotoxic ER stress (8, 28, 29). Consistent with our previous observations (10), we observed palmitate stimulation to increase *XBP1s* transcripts, indicative of activation of IRE1A (Supplemental Figure 2, A and B). Since palmitate can also activate the other UPR sensors, protein kinase R–like ER kinase (PERK) and activating transcription factor 6α (ATF6α) (30), we asked which of these pathways may regulate *S100A11*. We treated Huh7 cells with palmitate and specific pharmacological inhibitors for each of the UPR sensors and measured *S100A11* transcripts. The palmitate-stimulated Huh7 cells had maximum repression of *S100A11* transcripts in the presence of the IRE1A RNase-specific inhibitor (MKC8866) (Figure 2F), whereas a PERK inhibitor (GSK2606414) and ATF6α inhibitor (Ceapin-A7) did not repress palmitate-induced *S100A11* upregulation (Supplemental Figure 2C). We next employed a genetic approach, wherein we treated *Ire1a^–/–^* immortalized mouse hepatocytes (IRE1A-KO-IMH) with palmitate and noted lack of upregulation of *S100a11* transcript levels in comparison with WT-IMH controls (Supplemental Figure 2D). Thus, using complementary chemical and genetic approaches, we confirmed that the transcriptional upregulation of *S100A11* is IRE1A dependent.

Correspondingly, mass spectrometric analysis of the proteomic composition of the EVs collected from palmitate-stimulated IRE1A-KO-IMH cells (28) demonstrated S100A11 to be downregulated (Figure 2G), reinforcing the role of IRE1A in S100A11 upregulation and enrichment on lipotoxic EVs. To confirm these observations with a quantitative assay, we next used SPR-based measurement of S100A11 on EVs derived from palmitate- and vehicle-stimulated WT-IMH and IRE1A-KO-IMH cells. Palmitate stimulation enriched S100A11 abundance on the EVs derived from WT-IMH cells, while palmitate-stimulated IRE1A-KO-IMH cells secreted EVs that had lower levels of S100A11 (Figure 2H). Taking these results together, we inferred that lipotoxic ER stress regulates *S100A11* transcription and EV enrichment downstream of IRE1A signaling.

### Palmitate-induced lipotoxic ER stress epigenetically orchestrates S100A11 transcription.

To understand how IRE1A regulates *S100A11*, we assessed the role of XBP1s, which is the active spliced isoform of a transcription factor that acts as a key downstream mediator of the IRE1A signaling axis. We found a putative XBP1s consensus site in the *S100A11* promoter upstream of the transcription start site (TSS) in Huh7 cells (Supplemental Figure 3A). We designed ChIP primers that spanned the consensus site to assess XBP1s occupancy on the promoter and performed ChIP assays. ChIP–quantitative PCR (qPCR) demonstrated that palmitate stimulation in cell culture enriched XBP1s on the *S100A11* promoter relative to the isotype controls (Supplemental Figure 3B). We also assessed the human MASH livers by ChIP assay to verify XBP1s occupancy on the *S100A11* promoter. ChIP-qPCR demonstrated enrichment of XBP1s on the *S100A11* promoter in MASH livers compared with the control livers (Supplemental Figure 3C).

To investigate a functional, transcriptional outcome related to promoter occupancy, we used a dual reporter-based promoter luciferase assay. We cloned the promoter sequence of human *S100A11* (Supplemental Figure 3D) containing the XBP1s consensus site (31) into a pGL4.22 vector and assessed *S100A11* promoter activity with cotransfection of TK-*Renilla* plasmid in Huh7 cells. We also included a substitution mutant of the XBP1s consensus site in the assay. Palmitate treatment increased *S100A11* promoter activity modestly, yet statistically significantly, with respect to vehicle control (Supplemental Figure 3E). *S100A11* promoter mutant did not attenuate palmitate-induced *S100A11* promoter activity (Supplemental Figure 3E). Although we demonstrated the occupancy of XBP1s on the *S100A11* promoter by ChIP and activity in promoter reporter construct-based studies, the mutation studies suggested additional regulatory elements.

**Figure 3:**
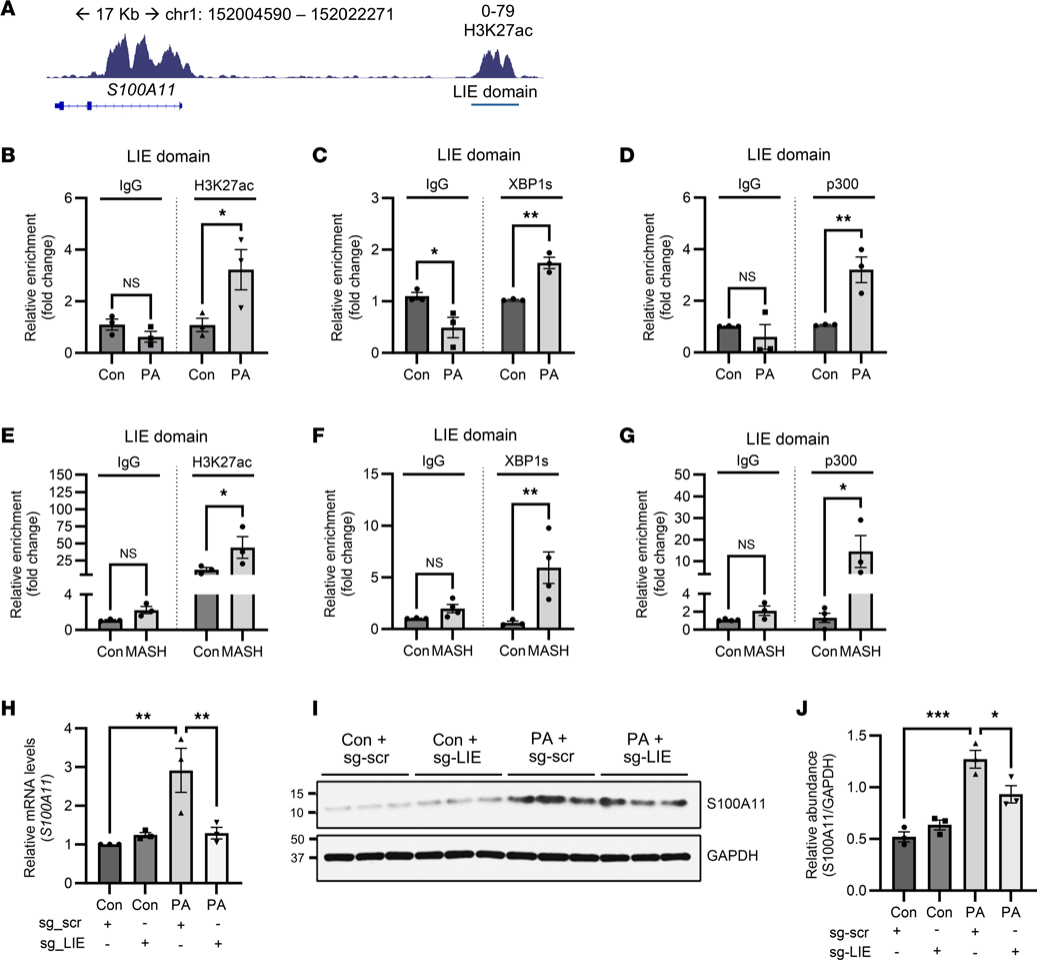
PA-induced lipotoxic ER stress epigenetically orchestrates S100A11 transcription. (**A**) Schematic representation of an H3K27ac peak (signal intensity of 0–78) on the promoter of the *S100A11* gene in Huh7 genome, using GEO dataset GSM2360939_1. The coordinates of the enhancer region are chromosome 1: 152019738–152021345. (**B**) ChIP-qPCR of H3K27ac on the LIE domain in Huh7 cells treated with 600 μM palmitate (PA) for 16 hours compared with vehicle-treated controls (*n* = 3 per group). Each group is normalized to respective IgG control. Two-way ANOVA with Šidák’s multiple-comparison test was used to compare 2 groups with 2 conditions. (**C** and **D**) ChIP-qPCR of XBP1s (**C**) and p300 (**D**) occupancy on the LIE domain in Huh7 cells treated with 600 μM PA for 16 hours compared with vehicle-treated controls (*n* = 3 per group). Each group is normalized to respective IgG control, which is repeated in each panel. Two-way ANOVA with Šidák’s multiple-comparison test was used to compare 2 groups with 2 conditions. (**E**–**G**) ChIP-qPCR of H3K27ac (**E**), XBP1s (**F**), and p300 (**G**) occupancy on the LIE domain in human MASH samples (*n* = 4) compared with controls (*n* = 4). Outliers were removed. Occupancy was normalized to input, then normalized to respective IgG negative control. Two-way ANOVA with Šidák’s multiple-comparison test was used to compare 2 groups with 2 conditions. (**H**–**J**) RNA (**H**) and protein (**I** and **J**) expression of *S100A11* in Huh7-dCas9-KRAB cells transiently transfected with LIE sgRNAs and treated with 600 μM PA for 20 hours compared with vehicle-treated controls (*n* = 3 per group). Molecular weight is noted in kilodaltons in the ladder. One-way ANOVA with Šidák’s multiple-comparison test was used to compare groups. *P < 0.05, **P < 0.01, ***P < 0.001.

Transcription is regulated at multiple levels, including chromatin remodeling, promoter activity, and enhancer function, which control DNA accessibility and gene expression. XBP1s is reported to interact with a histone lysine acetyltransferase, p300 (32, 33), which is a chromatin-modifying enzyme that regulates gene transcription (34–36). Thus, we interrogated histone acetylation downstream of lipotoxic ER stress in regulating the transcription of *S100A11*. Since H3K27ac symbolizes an active mark on the distal regulating enhancer elements (37), we reviewed the genomic region around the *S100A11* gene using publicly available H3K27ac ChIP-Seq datasets. We identified a 1.2 kb H3K27ac genomic region upstream of the TSS in Huh7 cells (Figure 3A), indicative of a putative enhancer element. We also noticed a similar H3K27ac domain within the same genomic coordinates in additional liver samples (Supplemental Figure 4A). In vitro analysis of this genomic region through ChIP-qPCR demonstrated an increase in H3K27ac of the enhancer region in palmitate-treated cells (Figure 3B), which we termed “lipotoxicity-influenced enhancer” (LIE).

Enhancer-mediated transcriptional regulation requires close proximity of the transcription factor and enhancer element (38). We therefore performed a ChIP assay to assess XBP1s interaction with the LIE domain. ChIP-qPCR analysis from palmitate-treated Huh7 cells showed increased enrichment of both XBP1s and p300 on the LIE domain (Figure 3, C and D), suggesting a functional coregulatory complex. To validate our in vitro findings, we analyzed human MASH livers and normal liver samples through ChIP-qPCR. MASH liver samples demonstrated enrichment of H3K27ac on the LIE domain and concomitant abundance of XBP1s as well as p300 on the LIE domain compared with the normal livers (Figure 3, E–G). As additional proof, we simultaneously treated Huh7 cells with palmitate and A485, a p300 inhibitor, and noted repression of palmitate-induced *S100A11* transcriptional upregulation on inhibition of p300 (Supplemental Figure 4B). Collectively, our data confirm the *S100A11* transcriptional program to be a multipartnered regulatory program comprising XBP1, p300, and the LIE domain (Supplemental Figure 4C).

To further establish biological relevance of the LIE element within palmitate-induced lipotoxicity, we used CRISPRi technology mediated by deactivated Cas9–Krüppel-associated box (dCas9-KRAB) (Supplemental Figure 4D). In this method (39), the recruitment of the dCas9-KRAB fusion protein, guided by the sgRNA, leads to the repression of enhancer-mediated epigenetic regulation. We compared the putative H3K27ac mark across publicly available ChIP-Seq GEO datasets for human hepatocyte and liver samples (Supplemental Figure 4A) and designed 2 sgRNAs targeting the LIE domain. We transiently transfected LIE-specific sgRNAs into the dCas9-KRAB–expressing Huh7 cell line (Supplemental Figure 4E) and stimulated the cells with palmitate. First, we assessed whether the LIE sgRNAs exhibited enhancer silencing by performing H3K27ac ChIP-qPCR. We observed that transient transfection of LIE sgRNAs reduced palmitate-induced H3K27ac activation mark on the LIE domain (Supplemental Figure 4F). We next interrogated whether LIE repression attenuated S100A11 transcription in lipotoxic hepatocytes. The LIE sgRNA–transfected cells failed to induce *S100A11* mRNA levels with palmitate treatment, with reduced expression in comparison with the palmitate-treated controls (Figure 3H). Palmitate-mediated increase in cytosolic S100A11 protein abundance was also attenuated in LIE sgRNA–transfected cells with respect to the controls (Figure 3, I and J).

Since our EV proteomics dataset had also identified S100A6 and S100A10 on lipotoxic EVs and several S100 family genes are clustered on the same chromosomal location (1q21.3 in humans and 3qF1–F2.1 in mice), we asked whether LIE regulated *S100A6* and *S100A10* transcripts. qPCR analysis demonstrated that LIE silencing did not affect the transcripts of *S100A6* and *S100A10* downstream of palmitate-induced lipotoxic ER stress (Supplemental Figure 5, A and B). Palmitate is reported to regulate induced gene expression via hyperacetylation (40). Hence, we asked whether palmitate-induced hyperacetylation regulated *S100A11* transcription via LIE activation. Although we observed palmitate stimulation to increase cellular acetyl-CoA levels (Supplemental Figure 5C), repression of the LIE domain in palmitate stimulation demonstrated attenuated *S100A11* transcripts and protein levels. These observations confirmed palmitate-induced *S100A11* upregulation via increased H3K27ac of the LIE domain to be epigenetically regulated by XBP1s-p300 complex and not an outcome of palmitate-induced increase in acetyl-CoA levels. Taken together, these results demonstrate that palmitate-induced lipotoxic ER stress mediates the transcriptional upregulation of *S100A11* via an epigenetically active enhancer, the LIE element.

### In vivo repression of the murine hepatic LIE attenuates steatohepatitis induced by choline-deficient, l-amino acid–defined high-fat diet.

We next wanted to extend our observations of a functional LIE element from a cellular model of palmitate-induced lipotoxicity to a murine model of steatohepatitis. Therefore, we asked whether repression of the LIE domain would attenuate hepatic S100A11 expression and thereby protect mice from developing MASH. To address this question, we reviewed publicly available H3K27ac ChIP-Seq datasets (Supplemental Figure 6A) from murine liver samples, to identify a mouse-specific LIE domain. We noticed an active H3K27ac peak, upstream of the TSS of the murine *S100a11* gene. We designed sgRNAs spanning this murine LIE region and screened them using dCas9-KRAB–expressing WT-IMH cells (Supplemental Figure 6B). We selected 2 sgRNA sequences that attenuated palmitate-induced *S100a11* upregulation (Supplemental Figure 6C) for subsequent in vivo studies. C57BL/6J mice with endogenous expression of dCas9-KRAB (41) were used for this study. Homozygous expression of the *dCas9-KRAB* cassette was verified by PCR (Supplemental Figure 6D). We next used commercially generated adeno-associated viral (AAV) vectors AAV8-LIE, which incorporated our validated sgRNAs, and AAV8-scramble (AAV8-scr) (Supplemental Figure 6E) for the murine studies.

**Figure 4:**
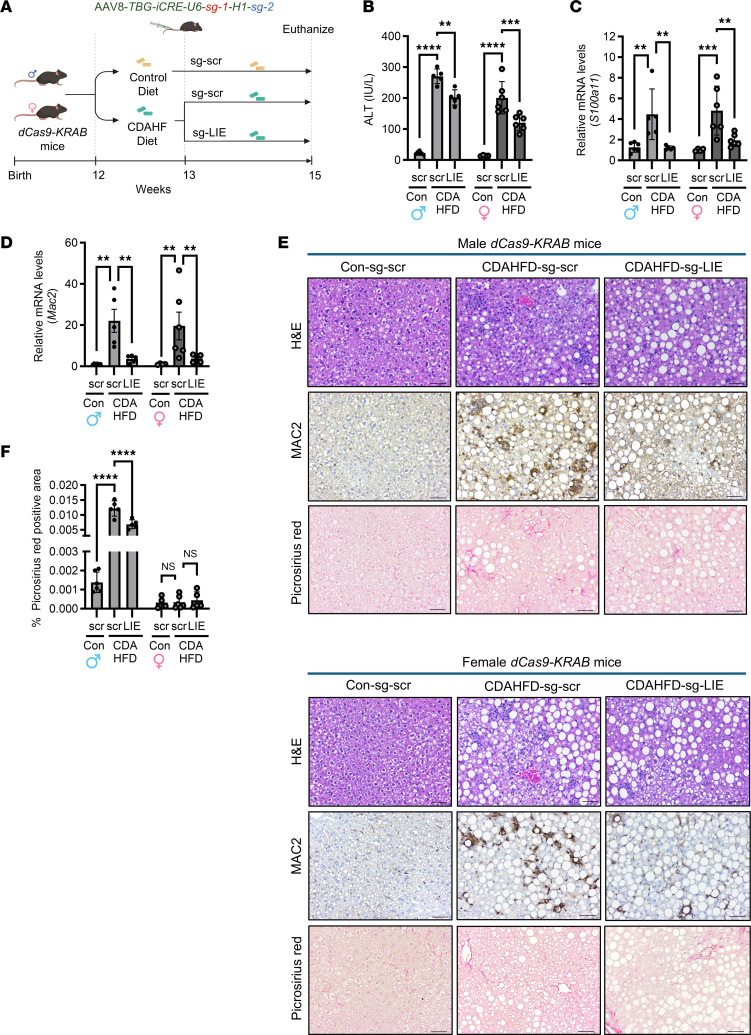
In vivo repression of the murine hepatic LIE lowers CDAHFD-induced steatohepatitis. (**A**) Schematic depicts the CDAHF diet regimen for the *dCas9-KRAB* mouse study. Male: con-scramble (*n* = 6), CDAHFD-scramble (*n* = 5), CDAHFD-LIE (*n* = 5); female: con-scramble (*n* = 4), CDAHFD-scramble (*n* = 6), CDAHFD-LIE (*n* = 6). (**B**) Serum ALT levels in male and female mice. Male mouse data points are represented as black circles and female mouse data points as gray circles. Two-way ANOVA with Šidák’s multiple-comparison test was used to compare 2 groups with multiple conditions. (**C** and **D**) Expression of *S100a11* (**C**) and *Mac2* (**D**) transcripts from the livers of AAV8-LIE–injected CDAHFD-fed mice. Two-way ANOVA with Šidák’s multiple-comparison test was used to compare 2 groups with multiple conditions. (**E**) Histological assessments of the liver sections via H&E staining (top row), MAC2 (galectin 3) staining (middle row), and Picrosirius red staining (bottom row) are demonstrated through representative images from evaluation of total number of samples as outlined in **A**. Scale bars: 50 μm. (**F**) Area of the Picrosirius red–positive collagen fibers per field was quantified using polarized microscopy. Each point represents 1 mouse with value averaged from 5 images (*n* = 4–6 mice per group). Two-way ANOVA with Šidák’s multiple-comparison test was used to compare 2 groups with multiple conditions. **P < 0.01, ***P < 0.001, ****P < 0.0001.

Our initial approach adopted the relatively rapid choline-deficient, l-amino acid–defined high-fat diet (CDAHFD) model of MASH with classic histological, biochemical, and signaling features of steatohepatitis (42, 43). Mice were placed on the CDAHFD for 1 week, after which they received either AAV8-LIE or AAV8-scr viral vectors at a dose of 1 × 10^12^ genome copies per mouse (Figure 4A). Mice were maintained on their respective diets for an additional 2 weeks prior to analysis. Phenotypically, the AAV8-LIE– or AAV8-scr–injected, CDAHFD-fed mice did not show a statistically significant difference in their body weight and liver/body weight ratios across the study groups (Supplemental Figure 7A). CDAHFD induced marked liver injury as measured by plasma levels of alanine transaminase (ALT) in AAV8-scr–injected mice. The CDAHFD-LIE group had lower ALT values compared with the CDAHFD-scr controls in both male and female mice (Figure 4B). We measured the expression of *iCre* transcripts as a readout of viral transduction and expression efficiency. The *iCre* expression was comparable across all virally transduced liver samples within each group (Supplemental Figure 7B). Viral transduction of AAV8-LIE was also verified by PCR amplifying a small amplicon of the AAV8-LIE plasmid followed by sequencing from AAV8-LIE–injected mouse livers (Supplemental Figure 7C). Concomitant with the ALT levels, CDAHFD-scr mouse livers had higher *S100a11* transcript levels compared with the control diet–fed mice transduced with AAV8-scramble (con-scr mice), while CDAHFD-LIE mice had an attenuation of *S100a11* transcripts compared with the CDAHFD-scr controls (Figure 4C). To investigate nonspecific effects of AAV8-LIE targeting, we assessed *S100a10* and *S100a13* genes, as they were located close to the LIE domain, and *BiP*, *Atf2*, and *Ager* genes, as they were associated with ER stress and S100A11 signaling. qPCR of the CDAHFD-fed mouse livers did not demonstrate a diet-induced increase in *S100a10* and *S100a13*, nor their repression (Supplemental Figure 7, D and E). Similar data were obtained for *BiP* and *Atf2* (Supplemental Figure 7, F and G). In contrast, *Ager* was induced by CDAHFD feeding, consistent with our prior studies (44), and was not repressed by AAV8-LIE (Supplemental Figure 7H). Thus, we did not observe nonspecific silencing of other genes in proximity, candidate UPR genes, or *Ager*, which is known to be upregulated in MASH.

With an aim to assess macrophage accumulation within the hepatic parenchyma, we quantified *Mac2* transcripts. mRNA abundance of *Mac2* was increased in CDAHFD-scr mouse livers, whereas CDAHFD-LIE had lower *Mac2* transcripts, suggesting a reduction in macrophage infiltration (Figure 4D). Correspondingly, H&E-stained liver sections demonstrated that CDAHFD-scr mouse livers developed steatosis and inflammation (Figure 4E). In contrast, CDAHFD-LIE mouse livers demonstrated no difference in steatosis but had fewer immune infiltrates (Supplemental Figure 7I), indicating that murine LIE repression reduces inflammatory cell accumulation. Liver MAC2 expression by immunohistochemistry (Figure 4E) confirmed CDAHFD-LIE mouse livers to have reduced macrophage infiltration compared with the CDAHFD-scr controls, both in male and female mice. Picrosirius red staining, indicative of fibrosis, demonstrated that CDAHFD-scr male mice had substantial pericellular fibrosis with a reduction in the CDAHFD-LIE male mouse livers by quantification of the Picrosirius red–positive area (Figure 4, E and F). Fibrogenic gene assessment demonstrated upregulation of *Timp1* and *aSMA* genes in the male CDAHFD-scr group compared with the con-scr, which were downregulated in the CDAHFD-LIE group (Supplemental Figure 7, J and K). *Col1a1* transcripts followed a similar trend, although the data points did not achieve statistical significance (Supplemental Figure 7L). Picrosirius red staining and fibrogenic gene expression studies showed no fibrosis in the female CDAHFD-scr or the CDAHFD-LIE mouse livers compared with the con-scr mouse livers (Figure 4, E and F, and Supplemental Figure 7, J–L). Taken together, our data demonstrate that repression of a murine LIE element on the *S100a11* promoter in a CDAHFD steatohepatitis model alleviated *S100a11* upregulation and steatohepatitis.

### In vivo repression of the murine hepatic LIE abrogates MASH induced by high-fat, -fructose, and -cholesterol diet.

**Figure 5:**
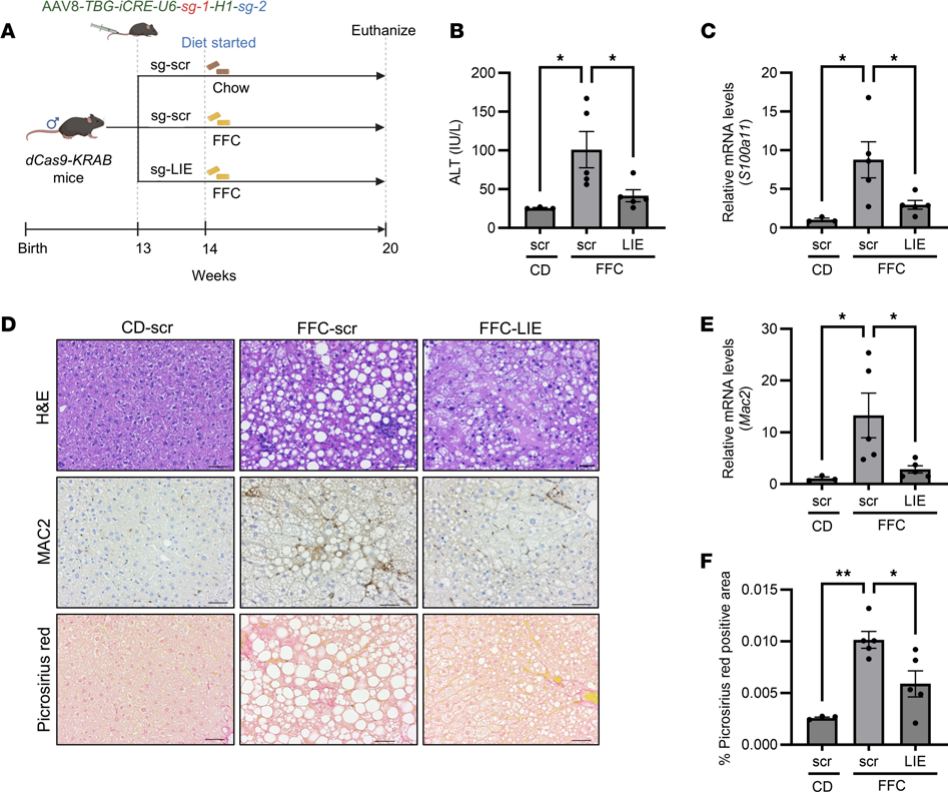
In vivo repression of the murine hepatic LIE lowers FFC diet–induced MASH. (**A**) Schematic depicting the FFC diet regimen for the *dCas9-KRAB* mouse study. CD-scramble (*n* = 3), FFC-scramble (*n* = 5), FFC-LIE (*n* = 5). (**B**) Serum ALT levels. One-way ANOVA with Šidák’s multiple-comparison test was used to compare 3 conditions. (**C**) Expression of *S100a11* transcript in the livers of AAV8-LIE–injected FFC-fed mice compared with the AAV8-scr–injected FFC control mice. One-way ANOVA with Šidák’s multiple-comparison test was used to compare 3 conditions. (**D**) Histological assessments of the liver sections via H&E staining (top row), MAC2 (galectin 3) staining (middle row), and Picrosirius red staining (bottom row) are demonstrated through representative images from a total number of slides as indicated in **A**. Scale bars: 50 μm. (**E**) Expression of *Mac2* transcripts in the livers of AAV8-LIE–injected FFC-fed mice compared with the AAV8-scr–injected FFC control mice. One-way ANOVA with Šidák’s multiple-comparison test was used to compare 3 conditions. (**F**) Area of the Picrosirius red–positive collagen fibers in the liver sections was quantified using polarized microscopy (*n* = 3–5 mice per group). Each point represents 1 mouse with values averaged from 5 images. One-way ANOVA with Šidák’s multiple-comparison test was used to compare 3 conditions. *P < 0.05, **P < 0.01.

In parallel, we tested whether the LIE was functional in vivo in the high-fat, -fructose, and -cholesterol (FFC) dietary model of MASH, which, in addition to the histological features of MASH, recapitulates condition-defining cardiometabolic risks of obesity, insulin resistance, and dyslipidemia (45). In this model, insulin resistance and steatosis develop by 4 weeks of feeding (45); hence, we chose a 6-week duration of FFC feeding. Mice were injected with AAV8 viral vectors before 6 weeks of FFC feeding, to equilibrate Cre recombinase protein and respective sgRNA abundance within the transduced hepatocytes, as depicted (Figure 5A). Liver/body weight ratio across the groups did not show a statistically significant difference (Supplemental Figure 8A). The ALT level was higher following 6 weeks of FFC-diet feeding in the mice injected with the scrambled construct (FFC-scr) compared with the rodent chow diet–fed mice (CD-scr) (Figure 5B). We noted reduction of ALT in the FFC-LIE group compared with the FFC-scr group (Figure 5B). The *iCre* expression was comparable across all virally transduced liver samples (Supplemental Figure 8B). Consistent with previous observations, the FFC-scr mice had upregulation of the expression of *S100a11* transcripts compared with CD-scr mice (Figure 5C). The FFC-LIE had attenuation of *S100a11* transcripts compared with the FFC-scr controls (Figure 5C). Assessment of *S100a10*, *S100a13*, *BiP*, *Atf2*, and *Ager* demonstrated no nonspecific effect of the AAV8-LIE within the FFC-LIE mouse livers (Supplemental Figure 8, C–G). Histological assessment of the liver sections demonstrated that 6 weeks of FFC caused extensive steatosis, with notable inflammatory foci, compared with the CD-scr controls (Figure 5D). FFC-scr mouse liver sections showed increased steatosis compared with the CD-scr mice, while AAV8-LIE–injected FFC-fed mice showed a visual redistribution from macrovesicular to mixed macrovesicular and microvesicular steatosis compared with the FFC-scr mice. BODIPY staining of the liver sections and quantification demonstrated a range of steatosis in the FFC-fed mice; however, the levels were comparable between the FFC-scr and FFC-LIE mouse livers (Supplemental Figure 8, H and I). Assessment of the inflammatory foci demonstrated reduction within the FFC-LIE mouse groups compared with FFC-scr controls (Figure 5D and Supplemental Figure 8J). Assessment of macrophage accumulation by immunohistochemistry and qPCR also confirmed an increase in inflammatory macrophage abundance in FFC-scr and reduction in FFC-LIE mice (Figure 5, D and E). Picrosirius red staining demonstrated an increase in pericellular collagen fibers in FFC-scr compared with the CD-scr groups, with a reduction in fibrosis in the FFC-LIE mouse livers (Figure 5, D and F). qPCR analysis of mouse livers showed upregulation of *Timp1*, *aSMA*, and *Mmp12* transcripts in FFC-scr mice compared with the CD-scr mice and downregulation in the FFC-LIE group to levels similar to those in CD-scr mice (Supplemental Figure 8, K–M). Taken together, our data demonstrate that repression of the murine LIE element on the *S100a11* genomic region in FFC diet–fed *dCas9-KRAB* mice led to a definitive reduction in liver injury, inflammation, and fibrosis following the repression of an *S100a11* transcriptional program.

Fibrosis can be attenuated as a result of etiological reversal or as a result of direct effects on hepatic stellate cells. While we noted a reduction in injury and inflammation in both MASH models treated with LIE sgRNA, consistent with etiological reversal, we also asked whether S100A11 may have direct effects on hepatic stellate cells via its cognate receptor, the receptor for advanced glycation end products (RAGE). We treated LX-2 cells (human hepatic stellate cell line) with recombinant human S100A11 protein and measured the expression of fibrogenic genes. S100A11 treatment upregulated *aSMA*, *TIMP1*, and *COL1A1* transcripts compared with respective controls (Supplemental Figure 9, A–C), suggesting a potential hepatocyte to stellate cell signaling axis mediated by S100A11 in MASH. In our recent work (44), we have reported the interaction between S100A11 and RAGE in macrophages to be vital in the proinflammatory response in MASH livers. Thus, taken together, our results demonstrate that S100A11, enriched on EVs and derived from lipotoxic hepatocytes under the control of a LIE element, can have pleiotropic outcomes through signaling responses in macrophages and hepatic stellate cells.

## Discussion

MASH is a hepatic manifestation of the metabolic syndrome and demonstrates hepatic lipotoxicity-induced sterile inflammation. DAMPs or alarmins secreted from lipotoxic hepatocytes regulate proinflammatory immune cell accumulation and activation in the liver. In this study, we report that (a) lipotoxic hepatocytes secrete S100A11 on EVs; (b) palmitate-induced hepatic lipotoxicity upregulates S100A11 via IRE1A/XBP1s signaling; (c) palmitate-induced lipotoxic ER stress epigenetically activates a distal regulatory element in the S100A11 genomic locus, which we have termed a LIE domain, demonstrated by enhanced H3K27ac marks; (d) dCas9-KRAB–mediated repression of the LIE lowers lipotoxicity-induced *S100a11* upregulation and associated inflammatory foci within the hepatic parenchyma of murine steatohepatitic livers; (e) there is an increase in S100A11-expressing hepatocyte-derived EVs in human MASH plasma samples; and (f) there is increased occupancy of p300 and XBP1s with H3K27ac on the LIE domain in human MASH liver samples along with an increase in *S100A11* transcript expression. These in vitro and in vivo observations present an integral crosstalk between lipotoxic ER stress and epigenetic regulation through a LIE domain that regulates the expression of S100A11.

Lipotoxicity is an essential step in hepatocellular injury in MASH, leading to both sublethal and lethal responses (46). Sublethal lipotoxic responses include organelle stress, such as ER stress, and the release of EVs. Studies have shown that EVs are qualitatively and quantitatively altered in MASH (10). Using an unbiased proteomics approach, this study identified the DAMP S100A11 to be enriched on lipotoxic EVs, in keeping with previous findings (11). Analysis of donor lipotoxic hepatocytes, from which the EVs originated, demonstrated lipotoxicity-induced transcriptional upregulation of *S100A11*. The role of ER stress in this transcriptional program was investigated owing to our earlier observations that lipotoxicity activates the ER stress response and that IRE1A activation drives the release of EVs from hepatocytes (10, 19).

Palmitate can activate all three UPR sensors; therefore, we used specific pharmacological inhibitors for each of the three sensors followed by confirmatory experiments in IRE1A-KO-IMH cells to demonstrate that deficiency of IRE1A attenuated the upregulation of *S100A11* transcripts in palmitate-treated hepatocytes. Mass spectrometric assessment of S100A11 abundance on EVs corresponded with the mRNA abundance. As mass spectrometry is not inherently quantitative, we confirmed these observations using SPR, which demonstrated an increase in S100A11-expressing EVs derived from WT-IMH cells and lack thereof in IRE1A-KO-IMH cells. Taken together, these results identified hepatic S100A11 as a readout of lipotoxic ER stress, transcriptionally upregulated by IRE1A signaling and released on EVs.

IRE1A activation leads to generation of spliced XBP1 (XBP1s), the transcriptionally active form of an evolutionarily conserved basic region/leucine zipper family transcription factor (47). We identified an XBP1s consensus motif on the promoter of *S100A11*, which demonstrated XBP1s occupancy and activity, yet it was not essential for the expression of *S100A11*, suggesting additional regulatory elements such as enhancers. It has been reported that XBP1s interaction with p300 enhances its transcriptional activity (32, 33), potentially via enhancers and superenhancers (48). Thus, we investigated a potential enhancer domain in the context of palmitate-stimulated XBP1s activation. Initial analysis of publicly available H3K27ac ChIP-Seq datasets identified an H3K27ac genomic region, marking a potentially active enhancer (37), upstream of the *S100A11* promoter. While H3K27ac was detected under the variable conditions of the published data sets, we empirically tested the relevance of this genomic region under lipotoxic conditions. In subsequent ChIP-qPCR studies we found this putative genomic region, which we named LIE, to be acetylated basally and demonstrate increased acetylation following palmitate stimulation. Furthermore, we confirmed the co-occupancy of XBP1s and p300 proteins on the LIE region. Using a dCas9-KRAB*–*mediated CRISPRi approach (41) and LIE-specific single-guide RNAs, we demonstrated that repression of the LIE attenuated palmitate-induced H3K27ac active marks on the LIE domain and *S100A11* transcripts. Based on our data, we propose that metabolic stress like lipotoxic ER stress causes increased recruitment of XBP1s and p300 to the LIE domain, leading to hyperactivity of the putative enhancer (LIE) and upregulation of S100A11. These data also suggest that lipotoxic ER stress hijacks an already active enhancer to mediate transcriptional upregulation of S100A11 in hepatocytes.

Enhancer elements function as a unique epigenetic platform for highly organized tissue-specific transcription. We aimed to repress the LIE region and assess hepatic *S100a11* levels in 2 well-established murine MASH models (42, 43, 45). Repression of the LIE domain led to attenuation of hepatic *S100a11* mRNA levels in AAV8-sg-LIE–injected mice in comparison with the AAV8-sg-scr–injected control mice, which was associated with a reduction in liver injury, inflammation, and fibrosis. There was a difference in the pattern of steatosis in FFC-LIE compared with FFC-scr mouse livers, with greater microvesicular steatosis, suggesting a role for S100A11 in hepatic steatosis. Indeed, S100A11 is known to promote lipogenesis via FOXO-1 (27), which may explain the observed differences in the pattern of steatosis. There was a reduction in inflammatory foci and macrophage accumulation in CDAHFD-LIE and FFC-LIE groups compared with the controls. Recently, we reported the role of RAGE-expressing macrophages in mediating liver proinflammatory macrophage accumulation (44). Our previous study postulated but did not identify a potential endogenous ligand for RAGE-expressing macrophages. In this study, we have identified that S100A11 is upregulated in MASH, and its inhibition attenuates macrophage accumulation, suggesting its role as an endogenous RAGE ligand in MASH.

In parallel, we observed a reduction in fibrosis and the expression of fibrogenic genes following LIE repression in male mice on MASH-inducing CDAHFD and FFC diets. Female CDAHFD-fed mice did not develop appreciable fibrosis, consistent with the reported resistance of female mice to CDAHFD-induced fibrosis (49). A reduction in injury and inflammation can lead to a reduction in liver fibrosis; however, we cannot exclude a direct effect of hepatocyte-derived S100A11-enriched EVs on hepatic stellate cells, as our studies with recombinant S100A11 protein and LX-2 cells demonstrated upregulation of fibrogenic genes. Thus, we believe that hepatocyte-derived S100A11 may have pleiotropic effects in the liver. The focus of the current study has been on defining the LIE domain in hepatocytes and its regulation of *S100A11* under lipotoxic conditions. Further studies with hepatic stellate cells are beyond the scope of the current work and could be examined in the future. Taken together, our study highlights a functional enhancer element, termed LIE, within the S100A11 regulatory framework that plays a key role in liver injury and inflammation associated with MASH progression.

To extend these observations to human MASH, we demonstrated that circulating hepatocyte-derived EVs are increased in MASH plasma compared with controls, consistent with the findings of several other groups (25, 50). In this study, we further demonstrate that S100A11 is enriched on each hepatocyte-derived EV in MASH plasma samples. Correspondingly, *S100A11* transcript is upregulated and there is an increase in the occupancy of p300 and XBP1s on the LIE domain along with an increase in H3K27ac. Thus, our findings confirm the relevance of lipotoxic ER stress–induced upregulation of S100A11 in MASH.

In conclusion, our study established a fundamental association between lipotoxic ER stress and epigenetics within the transcriptional program of *S100A11* in a lipotoxic hepatic milieu. We have defined an H3K27ac-marked active chromatin region upstream of the TSS of the *S100A11* gene as a LIE domain in lipotoxicity cell culture models, murine MASH, and human MASH. Targeted repression of this region using dCas9-KRAB methodology attenuated *S100A11* transcript levels and MASH. We present *S100A11* as a potentially druggable target to curb steatohepatitis and thereby reduce the severity and rate of MASH progression.

## Methods

### Sex as a biological variable.

We used 2 diet-induced murine MASH models for the study. Both male and female *dCas9-KRAB* mice were used for the CDAHFD feeding, because the disease penetrance is similar in both sexes. Only male *dCas9-KRAB* mice were used for the FFC-diet feeding, as female mice are resistant to FFC diet–induced obesity and MASH (45).

### Mouse studies.

Animal use was approved by the Institutional Animal Care and Use Committee (IACUC) of the Mayo Clinic and conducted in accordance with the NIH *Guide for the Care and Use of Laboratory Animals* (National Academies Press, 2011). *dCas9-KRAB*–expressing C57BL/6J-background mice (JAX 033066) were used for all experiments, were procured as a gift from Vijay Shah (Mayo Clinic, Rochester, Minnesota, USA), and are available from The Jackson Laboratory. Mice were housed in a 12-hour dark/12-hour light cycle. Mice were fed rodent CD or switched at 10–12 weeks of age to steatohepatitis-inducing diets. One group was fed with a CDAHFD for 3 weeks to induce steatohepatitis (42, 43), with the control group being fed the respective control diet. In our second model, male mice were fed with an FFC diet for 6 weeks to recapitulate human MASH (45). This diet has been previously described and characterized by us and others as having high fidelity to human MASH (10, 44, 45). For AAV injections, 1 × 10^12^ viral copies of AAV8-*TBG-iCre-U6-sgRNA1-H1-sgRNA2* (Vector Builder) were suspended in a total volume of 100 μL saline and injected into the tail vein. AAV8-LIE included LIE-specific sgRNA3 and sgRNA6 sequences, while AAV8-scr included 2 commercially generated scrambled sgRNAs. The sequence of each sgRNA is available in Supplemental Table 1. On completion of the study, mice were sacrificed, tissues were harvested, and platelet-poor plasma samples were analyzed for biochemistry parameters using a commercial veterinary chemistry analyzer (Abaxis, VetScan 2). Viral transduction was verified by PCR and confirmed by sequencing using the following steps. Briefly, a small piece of the mouse liver was processed using the Zymo DNA extraction kit to isolate the whole-liver genomic DNA. PCR primers were used to amplify specific amplicons from each vector backbone, and the PCR amplicon was gel-extracted and sequenced (Azenta). The DNA of the sgRNA for mouse LIE regions was checked for sequence similarity. AAV8-scr was also sequenced, but only the AAV8-LIE sequencing chromatogram is shown (Supplemental Figure 7C).

### Cell culture–based studies.

Huh7 cells were obtained from Sanjeev Gupta (and are available from the Japanese Collection of Research Bioresources, JCRB0403) and maintained in DMEM supplemented with 10% FBS and 1% penicillin-streptomycin antibiotics. WT-IMH (homozygous IRE-floxed) and IRE-KO-IMH cells were obtained from Randal Kaufman’s laboratory (Sanford Burnham Prebys, San Diego, California, USA) and have been previously described (11, 19, 20). IMHs were maintained in the same medium as above with an additional 1% non-essential amino acids. For palmitate-mediated lipotoxicity experiments, a stock of 80 mM palmitic acid (MilliporeSigma, P0500) was prepared in molecular-grade isopropanol and further diluted to a final concentration of 600 μM (for Huh7 cells), 400 μM (for IMH cells), and 400 μM (for PMH cells) in 10% FBS–containing medium and supplemented with 1% BSA. Inhibitor studies were performed with concurrent treatment with palmitic acid and inhibitor. For EV-based experiments, EV-free FBS was used in all treatment medium preparations. For transfection-based experiments, overnight-seeded 70%–80% confluent Huh7 cell monolayers were treated with respective plasmids complexed with GenJet reagent (SignaGen Laboratories), following the manufacturer’s protocol, and experiments were conducted within 24–48 hours. *dCas9-KRAB*–expressing Huh7 cells and IMH cells were prepared using the lentiviral plasmid (Addgene, 89567). sgRNAs (Integrated DNA Technologies) were transfected using Lipofectamine RNAiMAX (Invitrogen). sgRNA sequences used for human-LIE repression for in vitro studies are listed in Supplemental Table 1. Acetyl-CoA levels were measured using a commercially available assay, per the manufacturer’s protocol (MilliporeSigma, MAK039). Human stellate cell line LX-2 (a gift from Scott Friedman, Icahn School of Medicine at Mount Sinai, New York, New York, USA) were maintained in DMEM containing 10% FBS. LX-2 cells were treated with recombinant human S100A11 protein (SinoBiological, 11140-HNAE) as follows: 5 × 10^5^ LX-2 cells per well were seeded in a 6-well plate, and after overnight adherence, cells were serum-starved for 30 minutes in DMEM containing 1% free fatty acid–BSA. Cells were treated with 100 ng/mL of recombinant S100A11 for 4 hours (44). Cells were lysed in TRIzol for RNA isolation and gene expression analysis as described below.

### Primary mouse hepatocytes.

PMHs were isolated via portal vein perfusion using a 2-step collagenase-based protocol (51). The dissociated cells were further passed through a 70 μm filter and gently centrifuged at 50*g* for 2 minutes. The pelleted hepatocytes were purified using buffered Percoll in serum-free DMEM at 200*g* for 10 minutes. Cell viability was assessed, and PMHs were plated on collagen-coated dishes for experiments.

### Human samples.

Available biobanked human liver specimens and plasma samples, collected from MASH patients and obese normal patients as previously published (52, 53), and MASH and normal control samples from a Mayo Clinic Biobank were used after approval by the Mayo Clinic IRB and written informed consent for medical research. The diagnosis of MASH and histologically normal liver was established by expert pathologist review. In the MASH samples, fibrosis stage ranged from 1 to 3.

### EV isolation and proteomics.

EVs were collected from the cell culture supernatants using differential ultracentrifugation (10, 11). Cell supernatants were collected and centrifuged at 2,000*g* for 10 minutes to remove cellular debris. To isolate large EVs, the supernatant was centrifuged at 10,000*g* for 10 minutes, termed the 10K pellet, and the resultant supernatant was then transferred to an ultracentrifugation-compatible, open-top thick-wall polypropylene tube (Beckman Coulter) and centrifuged at 100,000*g* for 90 minutes to pellet small EVs, termed the 100K EVs. The pellets were washed in PBS, centrifuged at 100,000*g* for 2 hours, and resuspended in PBS until further use. 100K EVs were used for EV proteomics and immunogold labeling. EV proteomics was performed at the Mayo Clinic Proteomics Core Laboratory, as described previously (11). EVs were characterized by electron microscopy (EM) and immunogold staining using S100A11 antibodies with gold–protein A particles (10). EVs were prepared as whole mounts, labeled with immunogold, and negatively stained to visualize only the surface proteins. Objects were observed with JEOL 1400 electron microscope (10). For the S100A11 ELISA, both 10K and 100K EVs were isolated from Huh7 cell culture supernatants, and S100A11 levels were measured according to the manufacturer’s protocol (Invitrogen, EH397RB).

### SPR assay.

A Biosensing Instrument SPR (BI-2500) was used for analysis. Plasma samples from patients with MASH and healthy controls were processed by ultracentrifugation at 110,000*g* for 2 hours to isolate EVs, and isolated EVs were analyzed using a Nanoparticle Tracking Analyzer instrument (Malvern Panalytical, NS300) to measure EV concentration (19). Subsequently, MASH and control EVs were resuspended in 1× PBS buffer at the same concentration. Before analysis, a gold-coated SPR chip was functionalized with 11-mercaptoundecanoic acid (MUA; Sigma-Aldrich) in a manner described previously (54) to immobilize anti-ASGR2. EVs at a concentration of 2 × 10^9^ particles/mL in 1× PBS were infused into the SPR instrument at 10 μL/min flow rate for 10 minutes. Subsequently, the SPR chip was flushed with washing buffer (1× PBS) for 5 minutes to remove excess unbound EVs. SPR signals associated with EV capture represented the difference of the signal before the introduction of EVs and after washing buffer. Subsequently, anti-S100A11 (10 μg/mL in 1× PBS buffer; Supplemental Table 3) was injected into the SPR instrument for 5 minutes with a flow rate of 20 μL/min. The washing buffer was introduced for 5 minutes to remove excess antibodies. The SPR signal for S100A11 expression was taken as the difference of SPR signals before antibody and after washing. Subsequently, CYP2E1 and rabbit IgG antibodies were sequentially assayed. The binding results for each sample were obtained from 3 different (parallel) channels of the SPR instrument.

### Gene promoter assessments.

Publicly available hepatocyte/liver H3K27ac ChIP-Seq Gene Expression Omnibus (GEO) datasets (BigWig files) were read on the Integrative Genomics Viewer (IGV; https://igv.org/) platform and assessed for H3K27ac marks on the promoter of *S100A11*. Publicly available hepatocyte/liver ATAC files were also used to compare plausible transcription factor–binding regions on the enhancer domain to make specific primers that would enable a distinction between H3K27ac-based activation mark and XBP1s/p300 binding. XBP1s consensus (31) localization was accounted for during ChIP-qPCR primer preparation to enhance the possibility of greater reads from the ChIP eluates. For promoter activity, 800 bp of the human *S100A11* promoter upstream of the TSS was cloned into pGL4.22 plasmid (Promega, E6771) following conventional restriction digestion and ligation method. The XBP1s site was further mutated by substitution of CCACG to AAGAT (primer sequence for hS100A11 prom-M5 in Supplemental Table 2) using a Q5 Site-Directed Mutagenesis Kit (New England Biolabs, E0554S). The clones were sequenced to confirm cloning. A total of 1 μg of *S100A11* promoter–pGL4.22 construct (0.75 ng) was then cotransfected with TK-*Renilla* (0.25 ng) into Huh7 cells using GenJet reagent, in a 24-well plate. The cells were lysed after treatment using the Dual-Glo reagent (Promega), and relative luminescence units for both firefly and *Renilla* were recorded.

### ChIP assay.

To assess enrichment of XBP1s, p300, and H3K27ac mark on genomic regions, ChIP assay was performed using a ChIP-IT Express kit (Active Motif, 53008). Briefly, after respective treatments, cultured cells were formalin-fixed and weakly lysed to release intact nuclei. For human liver ChIP, intact nuclei were released from frozen tissue by mincing of approximately 100 mg of frozen tissue in 700 μL 1% formaldehyde followed by addition of 10× glycine solution to stop the reaction. This was pelleted and washed with PBS. The pellet was subsequently subjected to Dounce homogenization and incubated in 500 μL ice-cold Lysis Buffer for 30 minutes, after which nuclei were pelleted. Isolated nuclear pellets were lysed in the shearing buffer followed by optimized sonication-based chromatin fragmentation (25 Hz, cycle setting of 20 seconds on/20 seconds off, 5 times with a break of 1 minute between each cycle). An aliquot of the chromatin was cleared for total DNA content quantification, and an equal amount of chromatin was further processed for the ChIP assay. Appropriate isotype controls were also included alongside specific antibodies for ChIP pull-down. Elution of the antibody-bound specific chromatin after the required number of washes was performed following the Active Motif protocol. The eluate was then assessed for protein-interacting genomic regions via qPCR (primer sequences available in Supplemental Table 2), and the fold change between treatment groups for a specific antibody was calculated. Respective antibody concentrations and their catalog numbers are listed in Supplemental Table 3.

### qPCR analyses.

For in vitro experiments, cells were lysed in TRIzol, and total RNA was extracted using a commercially available kit (Zymo) following the manufacturer’s protocol. For mouse/human liver tissue samples, 5–10 mg of tissues were homogenized in TRIzol followed by RNA extraction using a kit (Zymo). One microgram of total RNA was further processed to prepare cDNA using an iScript cDNA preparation kit (Bio-Rad), following the manual. Diluted cDNA samples were assessed via qPCR, and relative fold change was calculated using the ΔΔCt method (55). One microliter of diluted cDNA was used as a template for reverse transcription PCR–based electrophoretic XBP1 splicing assessment (56). Three or more biological replicates were performed for each qPCR assay. 18S was used as loading control unless otherwise stated. Primer sequences are listed in Supplemental Table 2.

### Western blotting.

Cell or tissue lysates were prepared in RIPA buffer supplemented with a protease inhibitor cocktail. Total protein was quantified using BCA assay (Thermo Fisher Scientific). Twenty to thirty micrograms of protein was resolved in a 4%–20% gradient polyacrylamide SDS gel (Bio-Rad) and immunoblotted onto a 0.22 μm PVDF membrane using Tris-glycine transfer buffer. Nonspecific proteins were blocked with 5% nonfat dairy milk in TBS-T for 1 hour at room temperature and incubated with primary antibodies diluted in 5% BSA in TBS-T overnight at 4°C. The next day, membranes were washed and treated with respective secondary antibodies diluted in 5% nonfat dairy milk in TBS-T at room temperature. After another set of TBS-T washes, signals were visualized using ECL-based chemiluminescence and developed using film. Western blot densitometry was performed using ImageJ (NIH). Antibodies used for the study are listed in Supplemental Table 3.

### Histological studies.

H&E staining was performed by the Mayo Clinic Histology Core, Scottsdale, Arizona (10). For immunohistochemistry, formalin-fixed, paraffin-embedded tissue sections were stained with an antibody for MAC2 (Supplemental Table 3) using the ABC Immunostaining kit (Vector Laboratories) protocol. Fibrosis was assessed by Picrosirius red staining and quantified by the polarized light microscope method as described previously (44). BODIPY staining of mouse liver sections was performed as described previously (57).

### Statistics.

All experimental datasets represent mean ± SEM of data points obtained from 3 or more biological replicates. For 2 experimental groups, 2-tailed Student’s *t* test was used for statistical analyses in GraphPad Prism 9. Multiple treatment groups were analyzed using 1-way or 2-way ANOVA test, with Bonferroni’s or Šidák’s correction. A *P* value less than 0.05 was considered statistically significant. Outliers were identified using Grubbs’s test and removed from analysis.

### Study approval.

Mouse procedures were reviewed and approved by the Mayo Clinic IACUC. Biobanked human liver tissue samples and plasma samples were obtained following Mayo Clinic IRB approval and written informed consent from patients.

### Data availability.

A Supporting Data Values file containing all reported data values is included in the supplemental material. Unedited gel images are also included in the supplemental material. Publicly available H3K27ac ChIP-Seq datasets were accessed from the GEO database for liver cell line (GSM2360939_Huh7, GSM5911467_HepG2, and GSE168186_Hu1545), mouse liver (GSM5173253_A761, GSM5173254_A787, GSM4455320_Y.8.1, and GSM5404619_DKO_rep2), and human liver (GSM1112809_Adult_Liver4) samples.

## Author contributions

HM conceived the project. HM, PVD, and FHH designed the experiments. PVD, HLE, ASM, GP, YN, YY, DD, and TN conducted the experiments and interpreted the results. DC executed the SPR analysis supervised by AR and HM. JT helped with tail vein injections. HM provided all the reagents. PVD, HLE, and HM wrote the manuscript. All authors contributed to manuscript editing and provided a critical review and approval of the manuscript.

## Funding support

This work is the result, in part, of NIH funding and is subject to the NIH Public Access Policy. Through acceptance of this federal funding, the NIH has been given a right to make the work publicly available in PubMed Central.

- NIH grants DK111378 (to HM) and DK134661 (to AR).
- Mayo Foundation (to HM).
- Optical Microscopy Core and Clinical Core of the Mayo Clinic Center for Cell Signaling in Gastroenterology (NIH grant P30DK084567).
- Histology Core of the Mayo Clinic.
- American Liver Foundation Postdoctoral Research Fellowship Award 2024 (to PVD).

## Supplementary Material

Supplemental data

Unedited blot and gel images

Supporting data values

## Version 1 09/30/2025

In-Press Preview

## Version 2 12/01/2025

Electronic publication
