# Supplementary material for: ER stress upregulates S100A11 in steatohepatitis models via epigenetic modifications within the lipotoxicity-influenced enhancer
Source: J Clin Invest. 2025 Sep 30;135(23):e191074. doi: 10.1172/JCI191074 (PMC12646672; doi:10.1172/JCI191074)
Supplement: Supplemental data [file jci-135-191074-s053.pdf]

SUPPLEMENTARY FIGURES

Supplementary Figure 1:

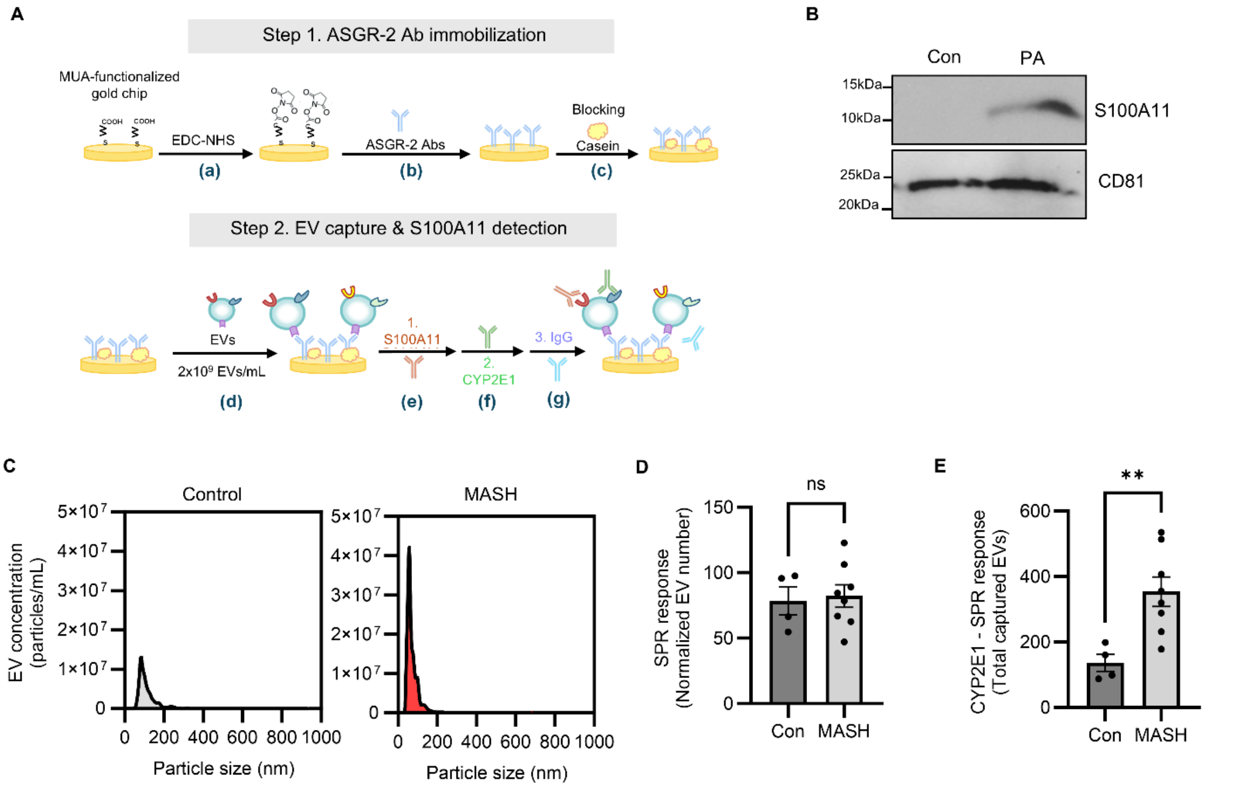

(A) Schematic representation of SPR and antibody-based quantification of EV-bound S100A11. **(B)** Representative western blot observation depicting S100A11 protein expression in EV lysates from Huh7 cells treated with 600 µM PA for 16 hours compared to vehicle-treated controls (n = 3 per group). **(C)** NTA-based quantification of EVs isolated from plasma samples of MASH (n = 8) and control (n = 4) patients. Representative peaks shown. **(D)** SPR response in resonance units (RU) for ASGR-2 mediated capture of hepatocyte-specific EVs from MASH (n = 8) and control (n = 4) plasma samples after EV number normalization. Student's unpaired two-tailed *t*-test was used to compare two groups. **(E)** SPR response in resonance units (RU) for CYP2E1 from S100A11-labelled, ASGR-2 captured hepatic EVs from MASH (n = 8) and control (n = 4) plasma samples. Student's two-tailed *t*-test was used to compare two groups. ** $P < 0.01$.

Supplementary Figure 2:

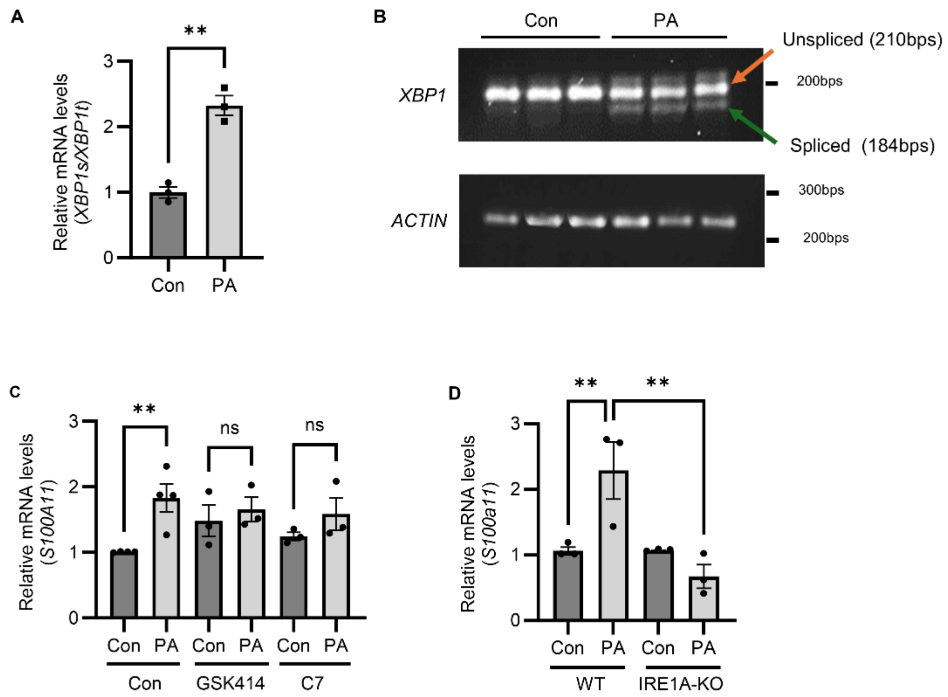

(A) Quantification of spliced *XBP1* mRNA levels normalized to total *XBP1* level in Huh7 cells treated with 600 µM PA for 16 hours compared to vehicle-treated control (n = 3 per group). Student's unpaired two-tailed *t*-test was used to compare two groups. **(B)** DNA electrophoresis gel image of *XBP1* mRNA splicing RT-PCR in Huh7 cells treated with 600 µM PA for 16 hours compared to vehicle-treated control (n = 3 per group). Unspliced XBP1 band is observed at 210bps and the spliced XBP1 band is at 184bps. **(C)** Expression of *S100A11* transcripts in Huh7 cells treated with 600 µM PA for 20 hours in the presence and absence of specific inhibition of PERK by treatment with 0.5 µM GSK2606414 (GSK414) or ATF6a by treatment with 0.25 µM Ceapin-A7 (C7) (n = 3 per group). Two-way ANOVA with Sidak multiple comparisons test was used to compare multiple groups with two conditions. **(D)** Expression of *S100a11* transcripts in WT-IMH and IRE-KO-IMH treated with 400 µM PA for 4 hours (n = 3 per group). Two-way ANOVA with Sidak multiple comparisons test was used to compare two groups with two conditions. ** $P < 0.01$.

Supplementary Figure 3:

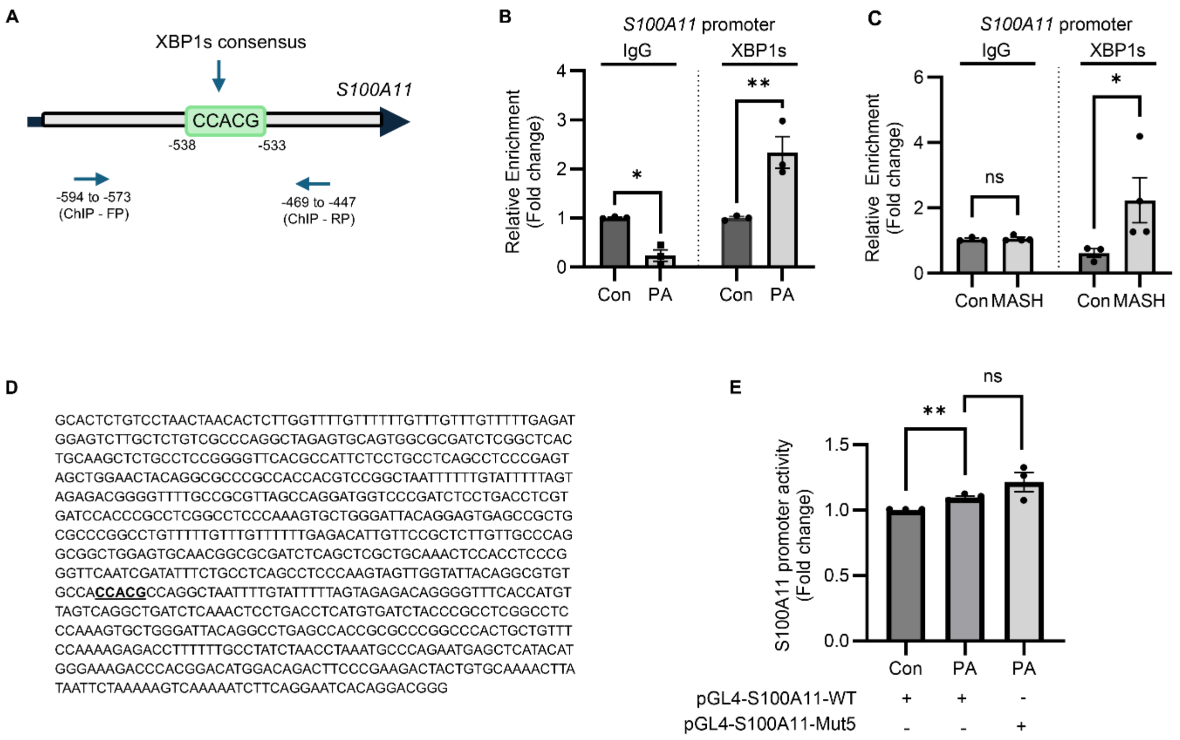

(A) Schematic representation of the human S100A11 promoter, with a putative XBP1s consensus site (CCACG) indicated upstream to the start of the first exon of transcript ENST00000271638.3. The location of the S100A11 promoter ChIP primers used in the study to score for XBP1s occupancy on chromatin is also indicated. **(B)** ChIP-qPCR of *S100A11* promoter region, using XBP1s-bound chromatin, in Huh7 cells treated with 600 µM PA for 16 hours compared to vehicle-treated control (n = 3 per group). Each group was normalized to the respective IgG control. Two-way ANOVA with Sidak multiple comparisons test was used to compare two groups with two conditions. **(C)** ChIP-qPCR analysis of *S100A11* promoter region, using XBP1s-bound chromatin, in human MASH livers compared to normal control livers (n = 4 per group). Outliers per Grubbs test were removed. Expression was normalized to input expression then normalized to respective IgG control. Two-way ANOVA with Sidak multiple comparisons test was used to compare two groups with two conditions. **(D)** Sequence of 800 base pairs from the human *S100A11* promoter that were inserted into pGL4.22 constructs for downstream promoter assays. The mutated bases representing the XBP1 consensus site are underlined and bolded. **(E)** Dual reporter-based assessment of relative *S100A11* promoter activity using wild-type and mutant *S100A11* promoter-pGL4.22 constructs and Renilla TK plasmids expressed in Huh7 cells treated with 600 µM PA for 20 hours compared to vehicle-treated controls. Student's unpaired two-tailed *t*-test was used to compare two conditions.

Supplementary Figure 4:

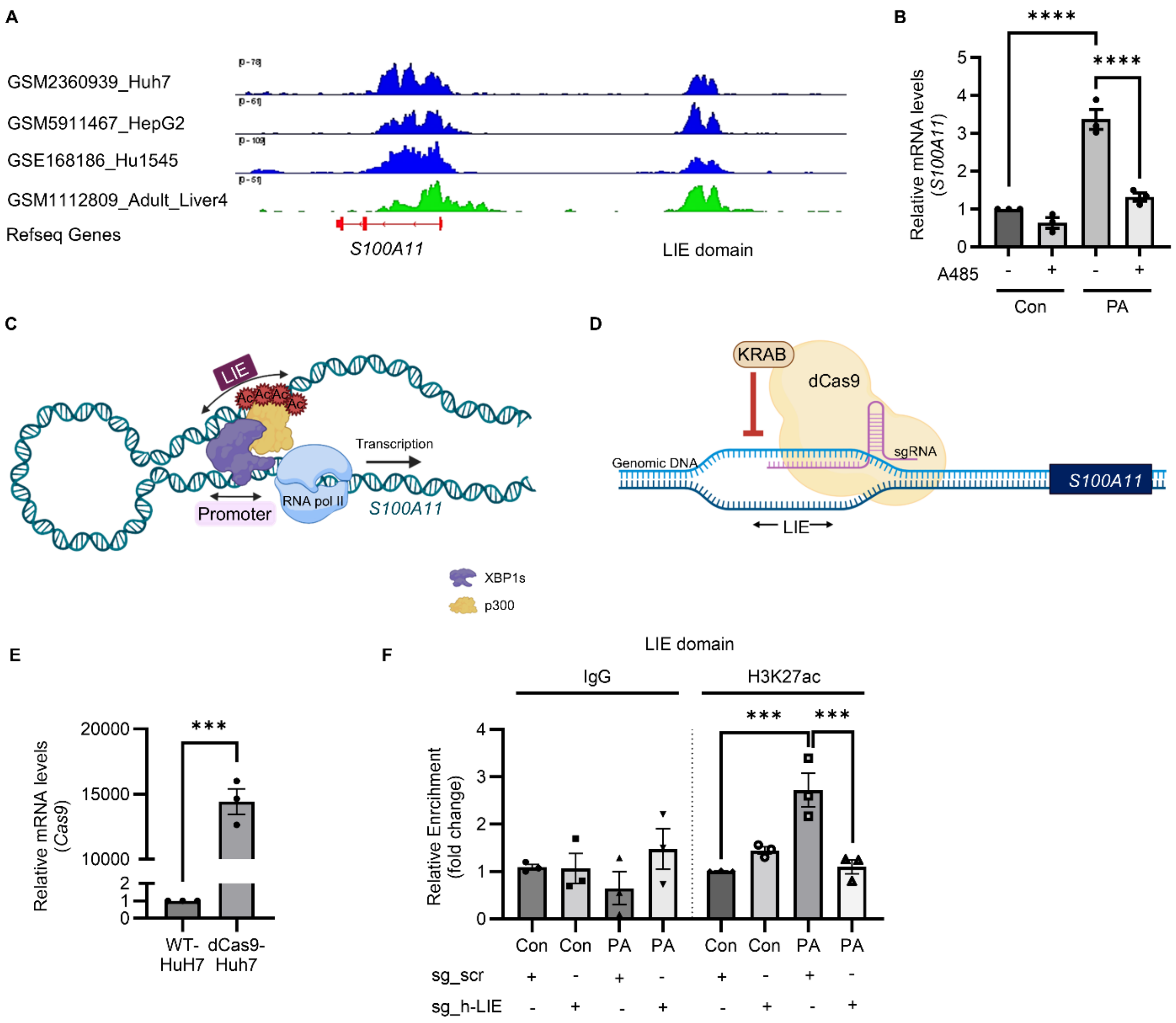

(A) Schematic representation of an H3K27acetylated peak on the promoter of *S100A11* gene, using publicly available H3K27ac-ChIP-seq datasets from Huh7, HepG2, and HU1545 cells (immortalized human hepatocytes), and human whole-liver. **(B)** Expression of *S100A11* transcript in Huh7 cells treated with 600 μM PA in combination with 1 μM A485 (p300 inhibitor) for 16 hours (n = 3 per group). Two-way ANOVA with Sidak multiple comparisons test was used to compare two groups with two conditions. **(C)** Proposed model of PA-induced lipotoxic ER stress-mediated upregulation of S100A11 transcription. **(D)** Schematic representation of the dCas9-KRAB CRISPRi model employed to repress epigenetic modifications on the LIE domain. **(E)** qPCR verification of *Cas9* expression in Huh7-dCas9-KRAB cells (n = 3 per group). Student's unpaired two-tailed *t*-test was used to compare groups. **(F)** ChIP-qPCR assessment of H3K27acetylation at the LIE domain in the presence or absence of human LIE sgRNA in Huh7-dCas9-KRAB cells treated with 400 μM PA for 20 hours (n = 3 per group). Each group was normalized to the respective IgG control. Two-way ANOVA with Sidak multiple comparisons test was used to compare multiple groups with multiple conditions. ***$P < 0.001$, **** $P < 0.0001$.

Supplementary Figure 5:

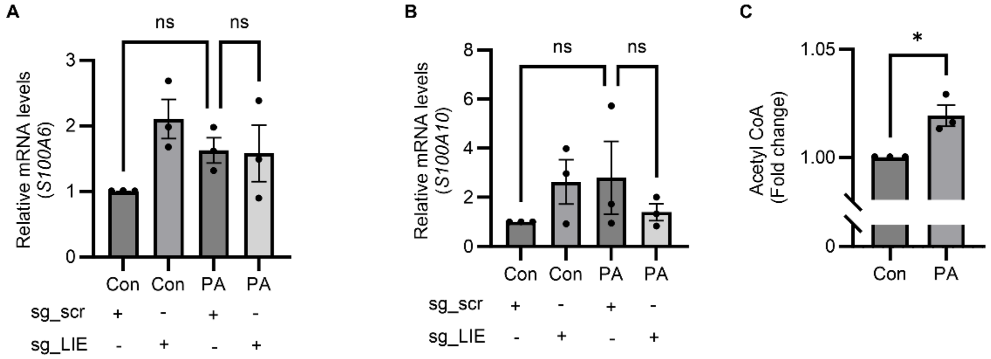

(A-B) Expression of (A) *S100A6* and (B) *S100A10* mRNA levels in both sg-scr transfected and sg-LIE transfected Huh7-dCas9-KRAB cells treated with 600 µM PA for 20 hours compared to vehicle-treated control (n = 3 per group). Two-way ANOVA with Sidak multiple comparisons test was used to compare two groups with two conditions. **(C)** Quantification of acetyl CoA levels in Huh7 cells treated with 600 µM PA for 20 hours compared to vehicle-treated controls (n = 3 per group). Student's unpaired two-tailed *t*-test was used to compare groups. *$P < 0.05$.

Supplementary Figure 6:

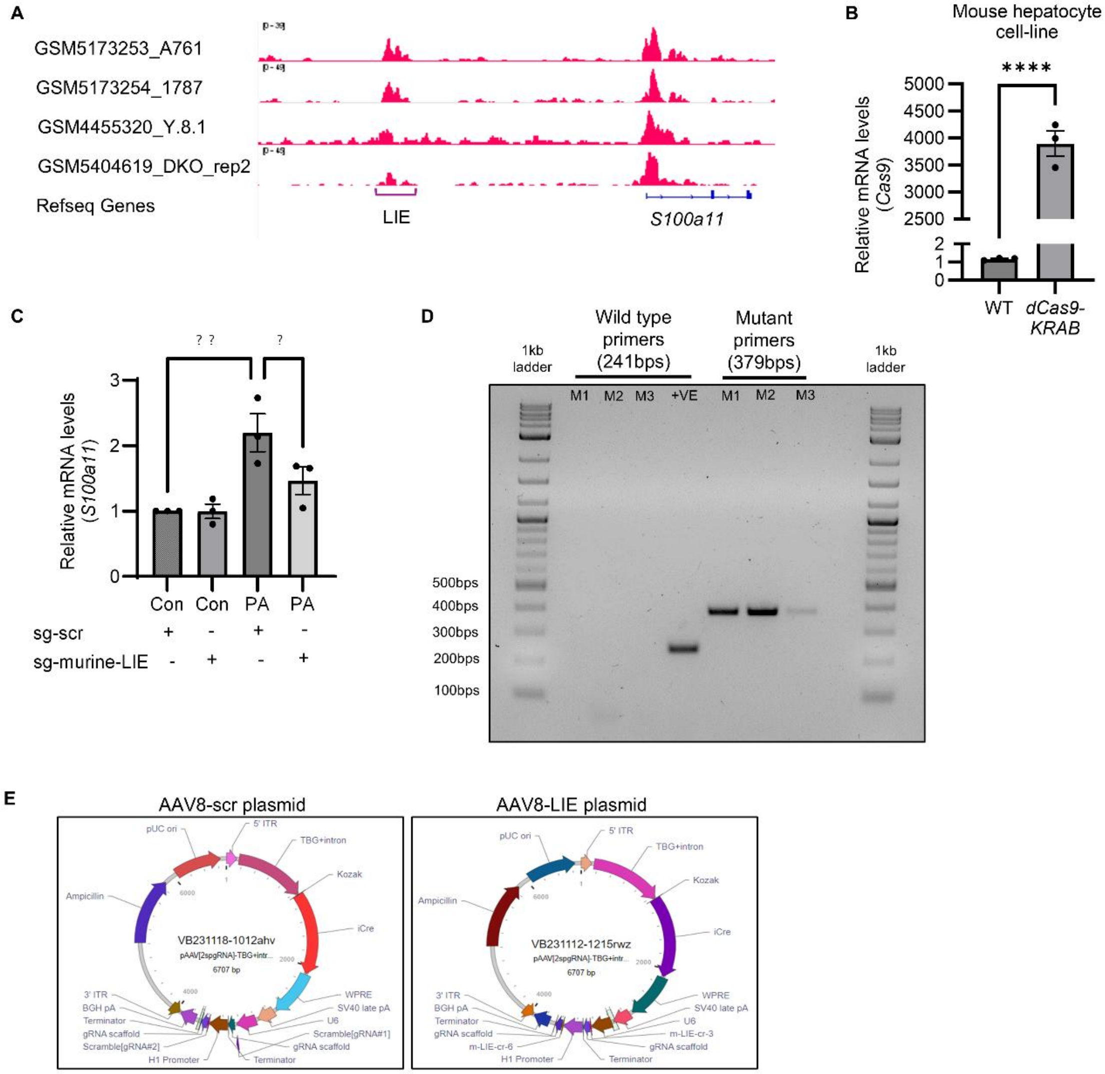

(A) Schematic representation of a H3K27acetylated peak on the promoter of mouse *S100a11* gene using publicly available H3K27ac ChIP-seq from mouse liver samples. **(B)** qPCR verification of *Cas9* expression in dCas9-KRAB-WT-IMH cells compared to WT-IMH controls (n = 3 per group). Student's unpaired two-tailed *t*-test was used to compare groups. **(C)** Expression of *S100a11* mRNA levels in dCas9-KRAB-WT-IMH cells treated with 400 μM PA for 16 hours compared to vehicle-treated controls (n = 3 per group). Two-way ANOVA with Sidak multiple comparisons test was used to compare two groups with two conditions. **(D)** PCR verification of the homozygous dCas9-KRAB breeder cage mice, following Jackson Laboratory's protocol. Mutant primers amplify the *dCas9-LSL* cassette region, while wild-type primers amplify uninterrupted *ROSA* region (Mutant = 379 bp, Heterozygote = 241 bp and 379 bp, Wild type = 241 bp). Labels M1 to M4 indicate the 4 mice used for genotyping. **(E)** Vector map of the AAV8 constructs customized from the Vector Builders company confirms the appropriate sgRNA sequences within the respective AAV8-viral backbones. * $P < 0.05$, ** $P < 0.01$, **** $P < 0.0001$.

Supplementary Figure 7:

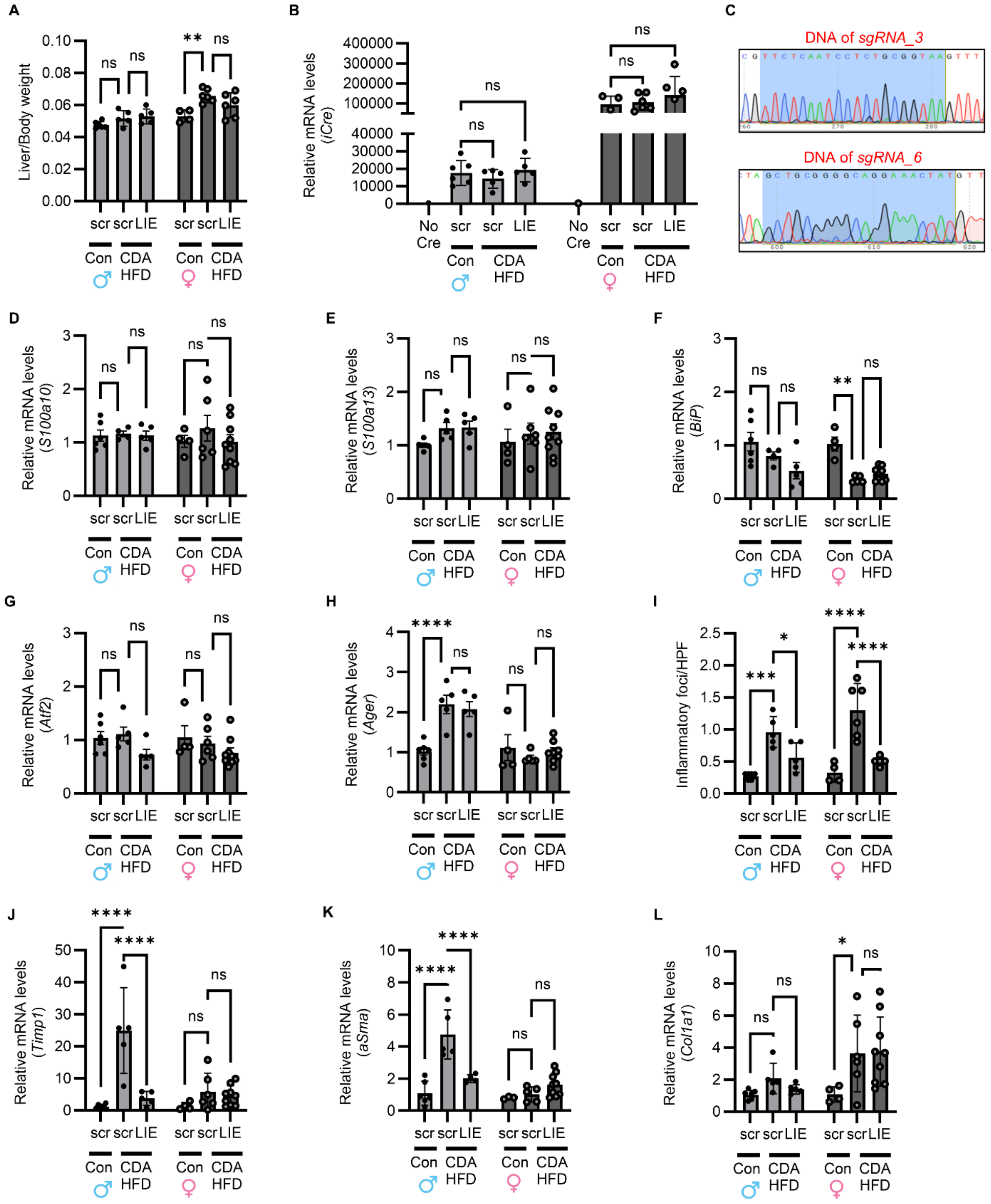

(A) Liver to body weight ratio of the CDAHFD-fed *dCas9-KRAB* male and female mice cohorts (n = 4-6 per group). **(B)** Relative iCre transcript abundance in the whole-livers of the CDAHFD-fed *dCas9-KRAB* cohorts, analyzed using qPCR and normalized to respective no Cre control. **(C)** Sequencing the PCR inserts amplified using specific primers for the AAV8-LIE plasmid verified

the significant transduction and abundance of the *sgRNA-3* and *sgRNA-6* sequences within the mouse liver. **(D-H)** qPCR-based quantification of non-specific AAV8-LIE targeting presented no significant effect on genes near to LIE region, **(D)** *S100a10* and **(E)** *S100a13*, and distant genes **(F)** *BiP,* **(G)** *Atf2, and* **(H)** *Ager* (n = 4-6 mice per group). **(I)** Ten fields per mouse were quantified for the inflammatory foci in H&E staining at ×20 magnification. Each point represents one mouse with value averaged from 10 images (n = 4-6 mice per group). **(J-L)** Fibrogenic gene **(J)** *Timp1,* **(K)** *aSMA,* and **(L)** *Col1a1* expression in the whole-livers of the CDAHFD-fed *dCas9-KRAB* cohort. For all panels, two-way ANOVA with Sidak multiple comparisons test was used to compare two groups with multiple conditions. *$P < 0.05$, **$P < 0.01$, ***$P < 0.001$, **** $P < 0.0001$.

Supplementary Figure 8:

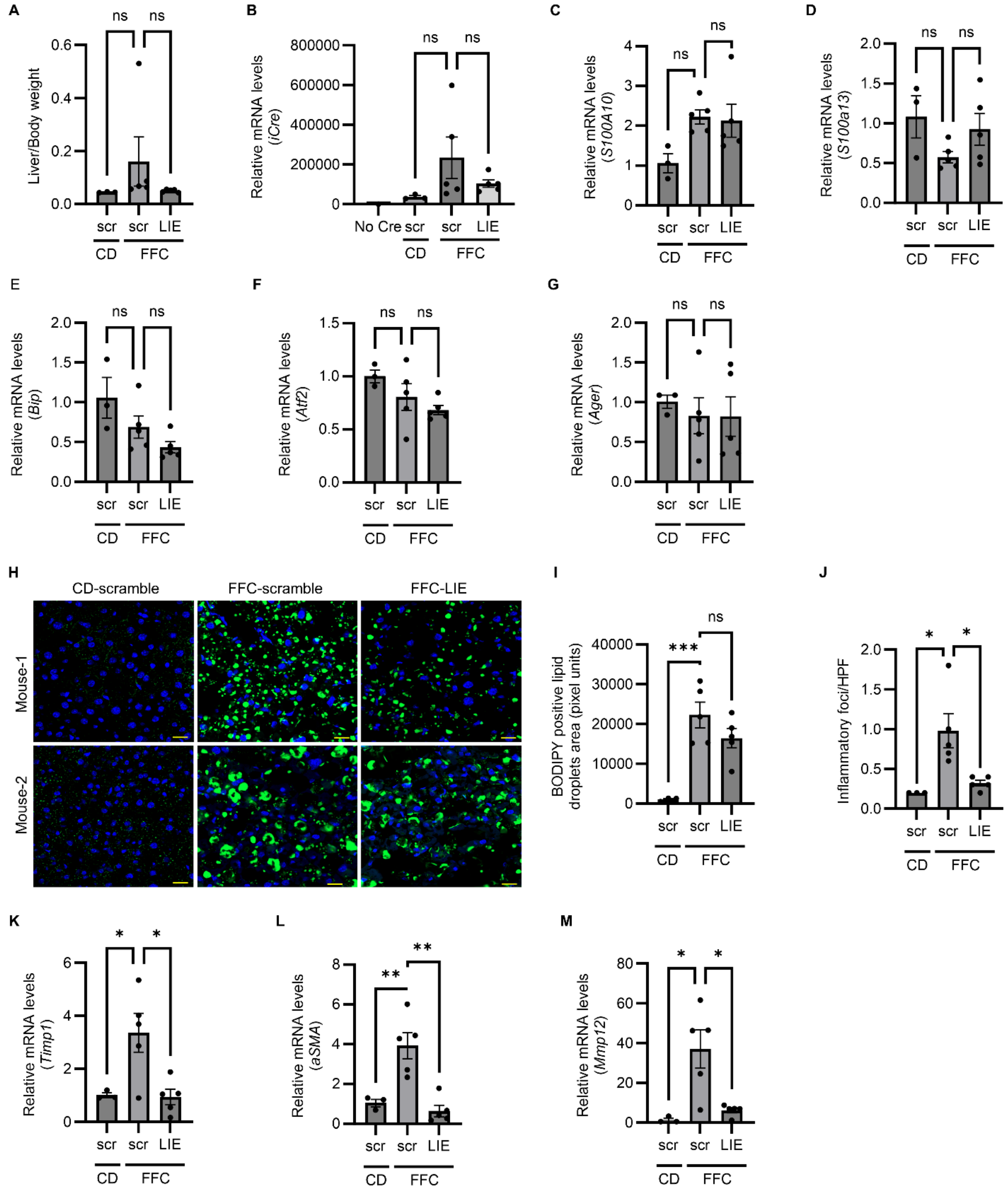

(A) Liver to body weight ratio of the FFC-fed *dCas9-KRAB* male mice cohorts (n = 3-5 mice per group). **(B)** Relative *iCre* transcript abundance in the whole-livers of the FFC-fed *dCas9-KRAB* cohorts (n = 3-5 mice per group). **(C-G)** qPCR-based quantification of non-specific AAV8-LIE targeting presented no significant effect on genes near to LIE region, **(C)** *S100a10* and **(D)**

S100a13, and distant genes **(E)** *BiP,* **(F)** *Atf2,* and **(G)** *Ager* (n = 3-5 mice per group). **(H)** Liver sections of the FFC-fed cohort were scored for steatosis using BODIPY staining. Images of two best representative mice per group have been depicted. **(I)** Relative area of BODIPY stained lipid droplets were quantified across all mouse livers from the cohort using ImageJ. Each data point represents one mouse averaged over 3 images (n = 3-5 mice per group). **(J)** Ten fields per mouse were quantified for the inflammatory foci in H&E staining at ×20 magnification. Each point represents one mouse with value averaged from 10 images (n = 3-5 mice per group). **(K-M)** Fibrogenic gene (K) *Timp1,* (L) *aSMA,* and (M) *MMP12* expression in the whole livers (n = 3-5 mice per group). For all panels, one-way ANOVA with Sidak multiple comparisons test was used to compare multiple groups. *$P < 0.05$, **$P < 0.01$, ***$P < 0.001$.

Supplementary Figure 9:

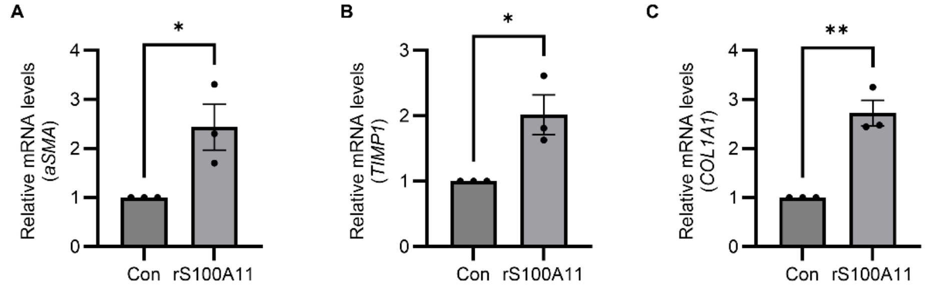

(A-C) Expression of fibrogenic genes **(A)** *aSma*, **(B)** *Timp1*, and **(C)** *Col1a1* in LX-2 cells treated with human recombinant S100A11 (100 ng/µl) for 4 hours compared to untreated controls. For all panels, student's unpaired two-tailed *t*-test was used to compare two groups. $*P < 0.05$, $**P < 0.01$.

SUPPLEMENTARY TABLES

Table-1: sgRNA sequences used in the study.

sgRNA	Sequence
h-LIE-sg_1	CTGTATTAGAAATAATAGGC
h-LIE-sg_3	TCGGGATCTGGTCGCCTGTG
m-LIE-sg_3	TTCTCAATCCTCTGCGGTAA
m-LIE-sg_6	GCTGCGGGGCAGGAAACTAT
Scr-sg_1	GTGTAGTTCGACCATTCGTG
Scr-sg_2	GTTCAGGATCACGTTACCGC

Table-2: List of Primers

**Primer**	**Sequence**
h-S100A11-F	CCAGAAGTATGCTGGAAAGGATG
h-S100A11-R	CATCATGCGGTCAAGGACACCA
h-XBP1s-F	GCTGAGTCCGCAGCAGGT
h-XBP1s-R	CTGGGTCCAAGTTGTCCAGAAT
h-XBP1t-F	TGAAAAACAGAGTAGCAGCTCAGA
h-XBP1t-R	CCCAAGCGCTGTCTTAACTC
h-S100A11-Ch-F	TCAATCGATATTTCTGCCTCA
h-S100A11-Ch-R	TAGATCACATGAGGTCAGGAGT
h-LIE-S1-Ch-F	GGAACTCTCTCGCTCCAATC
h-LIE-S1-Ch-R	TCCTCTCTTCCTCTAGACAC
h-LIE-S2-Ch-F	GGTCTGTGACTTTCCAGTTAC
h-LIE-S2-Ch-R	GCAGAGTGTGCCTTTCTGC
h-S100A6-F	AGCACACCCTGAGCAAGAAGGAG
h-S100A6-R	CTCCTGGAAGTTCACCTCCTGGT
h-S100A10-F	AACAAAGGAGGACCTGAGAGTAC
h-S100A10-R	CTTTGCCATCTCTACACTGGTCC
h-Timp1-F	CTTCTGGCATCCTGTTGTTG
h-Timp1-R	GGTATAAGGTGGTCTGGTTG
h-aSMA-F	GGAGATCACGGCCCTAGCAC
h-aSMA-R	AGGCCCGGCTTCATCGTAT
h-COL1A1-F	TGTGAGGCCACGCATGAG
h-COL1A1-R	CAGATCACGTCATCGCACAA
*h-S100A11 prom-Kpn-F**	CGGGGTACCGCACTCTGTCCTAACTAACACTCTT
*h-S100A11 prom-HindIII-R**	CCCAAGCTTCCCGTCCTGTGATTCCTGAAGATT
*h-S100A11 prom-M5-F**	GTGCCAAAGATCCAGGCTAATTTT
*h-S100A11 prom-M5-R**	ACGCCTGTAATACCAACTACTT
m-S100A11-F	AAGTACAGCGGGAAGGATGGA
m-S100A11-R	ATGCGGTCAAGGACACCAG
m-S100A11-Ch-F	AAAGAATTCAATAGGGGCAAA
m-S100A11-Ch-R	GCAGTATTTAGTTTTGAGCCAT
m-Mac2-F	TGGGCACAGTGAAACCCAAC
m-Mac2-R	TCCTGCTTCGTGTTACACACA
m-Timp1-F	AGGTGGTCTCGTTGATTTCT
m-Timp1-R	GTAAGGCCTGTAGCTGTGCC

m-aSma-F	GTCCCAGACATCAGGGAGTAA
m-aSma-R	TCGGATACTTCAGCGTCAGGA
m-Mmp12-F	CTGCTCCCATGAATGACAGTG
m-Mmp12-R	AGTTGCTTCTAGCCCAAAGAAC
m-Col1a1-F	GCTCCTCTTAGGGGCCACT
m-Col1a1-R	CCACGTCTCACCATTGGGG
m-S100a10-F	TGGAAACCATGATGCTTACGTT
m-S100a10-R	GAAGCCCACTTTGCCATCTC
m-S100a13-F	AACTGCCTCATTTGCTCAAGG
m-S100a13-R	AGTCTCCAGTATTCACTGAACCT
m-Bip-F	ACTTGGGGACCACCTATTCCT
m-Bip-R	ATCGCCAATCAGACGCTCC
m-Atf2-F	CTTCCTCTCCTCAACCAGTCCA
m-Atf2-R	GAGTCCTAACCAATCCACTGCC
m-Ager-F	ACAGGCTCTGTGGGTGAGTCT
m-Ager-R	CTGACTGATTCAGCTCTGCAC
iCre-F	AGAAGAAGAGGAAAGTCTCCAACCT
iCre-R	AACCATTTCCTGTTGTTCAGCTTGC
h/m-18S-F	CGCTTCCTTACCTGGTTGAT
h/m-18S-R	GAGCGACCAAAGGAACCATA
AAV8-LIE-FP*	CCTCTACAAATGTGGTACAAGTTTG
AAV8-LIE-RP*	AGAATGACACCTACTCAGACAATG
AAV8-scr-FP*	GACAAACCACAACTAGAATGCAG
AAV8-scr-RP*	GTGAATTCGGTACCTCTAGAC

(Note: Ch- ChIP primers, *- Cloning/Sequencing primers)

Table-3: List of Antibodies

Target	Source	Use
S100A11	Proteintech, Cat:10237-1-AP	Western blotting and SPR – human samples
S100A11	Thermo, Cat: PA5-102787	SPR – mouse samples
GAPDH	Millipore, Cat: MAB374	Western blotting
CD81	Santa Cruz Biotechnology, Cat: sc-166029	Western blotting
ASGR2	R&D systems, Cat: MAB9970	SPR
CYP2E1	CYP450-GP, Cat: Hu-A002	SPR
XBP1s	Cell Signaling Technology (E9V3E), Cat: #40435	ChIP – mouse samples<br>2µg antibody per assay
XBP1s	Biolegend, Clone-9D11A43, Cat: 658802	ChIP – human samples<br>2µg antibody per assay
H3K27ac	Diagenode, Cat: C15410196	ChIP – all samples<br>1µg antibody per assay
p300	Diagenode, Cat: C15200211	ChIP – all samples<br>1µg antibody per assay
Mac2	Invitrogen, Cat: 14-5301-82	IHC
Rabbit IgG	Sigma, Cat: I5006-10MG<br>Cell Signaling Technology, Cat: #2729	ChIP control
Mouse IgG	Cell Signaling Technology (G3A1), Cat: #5415	ChIP control
