# Supplementary material for: ER stress upregulates S100A11 in steatohepatitis models via epigenetic modifications within the lipotoxicity-influenced enhancer
Source: J Clin Invest. 2025 Sep 30;135(23):e191074. doi: 10.1172/JCI191074 (PMC12646672; doi:10.1172/JCI191074)

Full unedited blot for Figure 2D

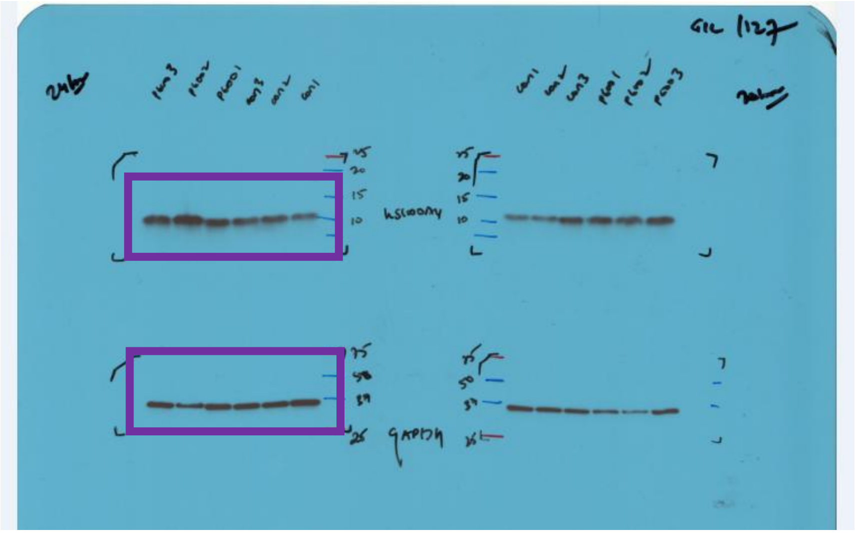

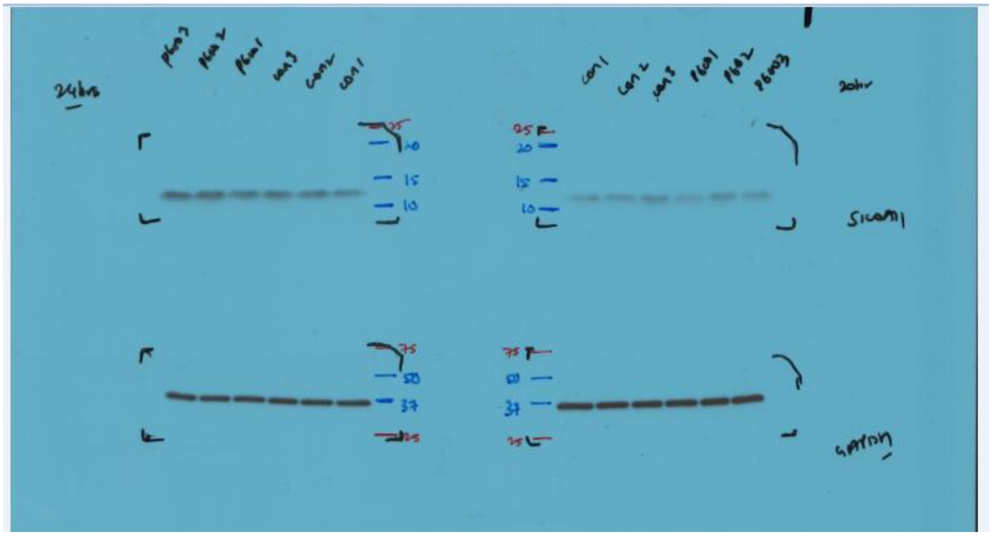

Full unedited blot for Figure 3I

TRIAL-1

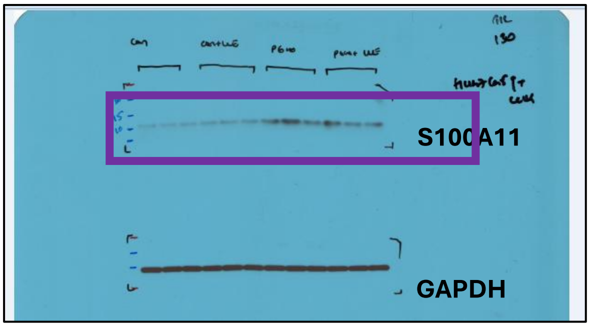

Lower exposure

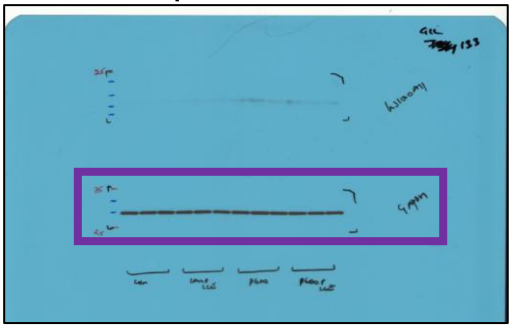

TRIAL-2

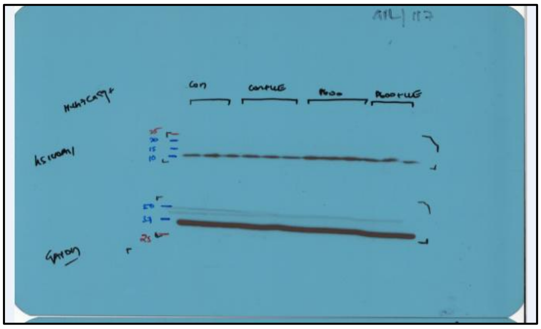

Lower exposure

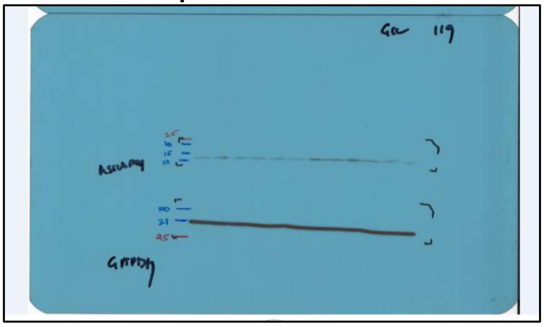

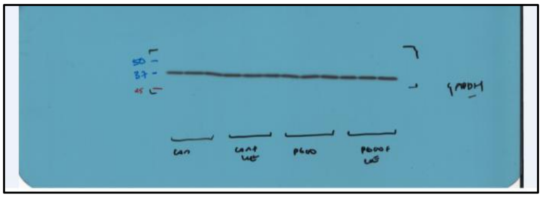

Full unedited blot for Supplemental Figure 1B

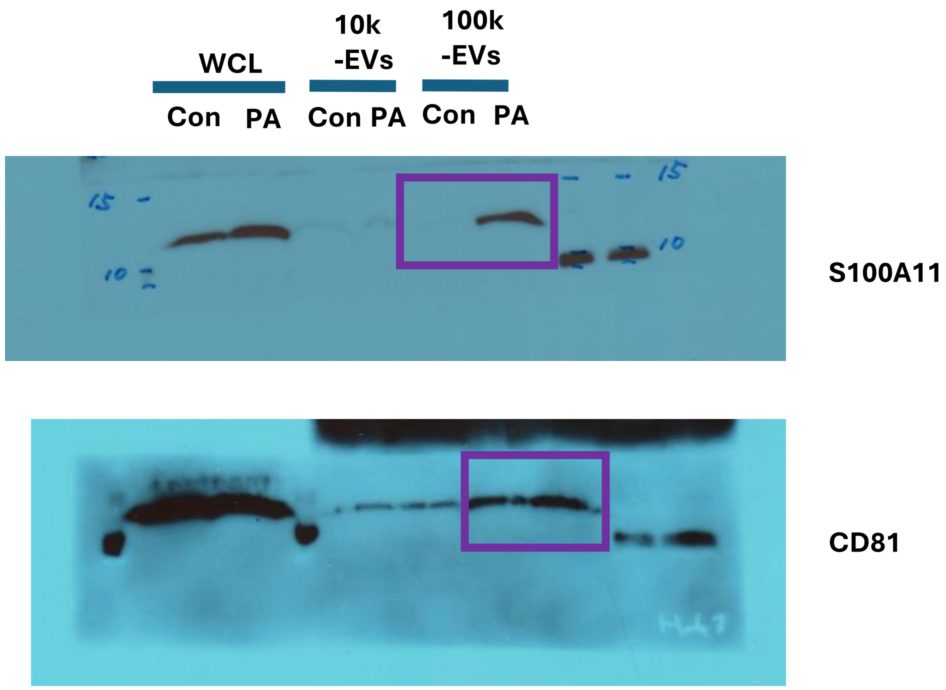

Full unedited gel for Supplemental Figure 2B

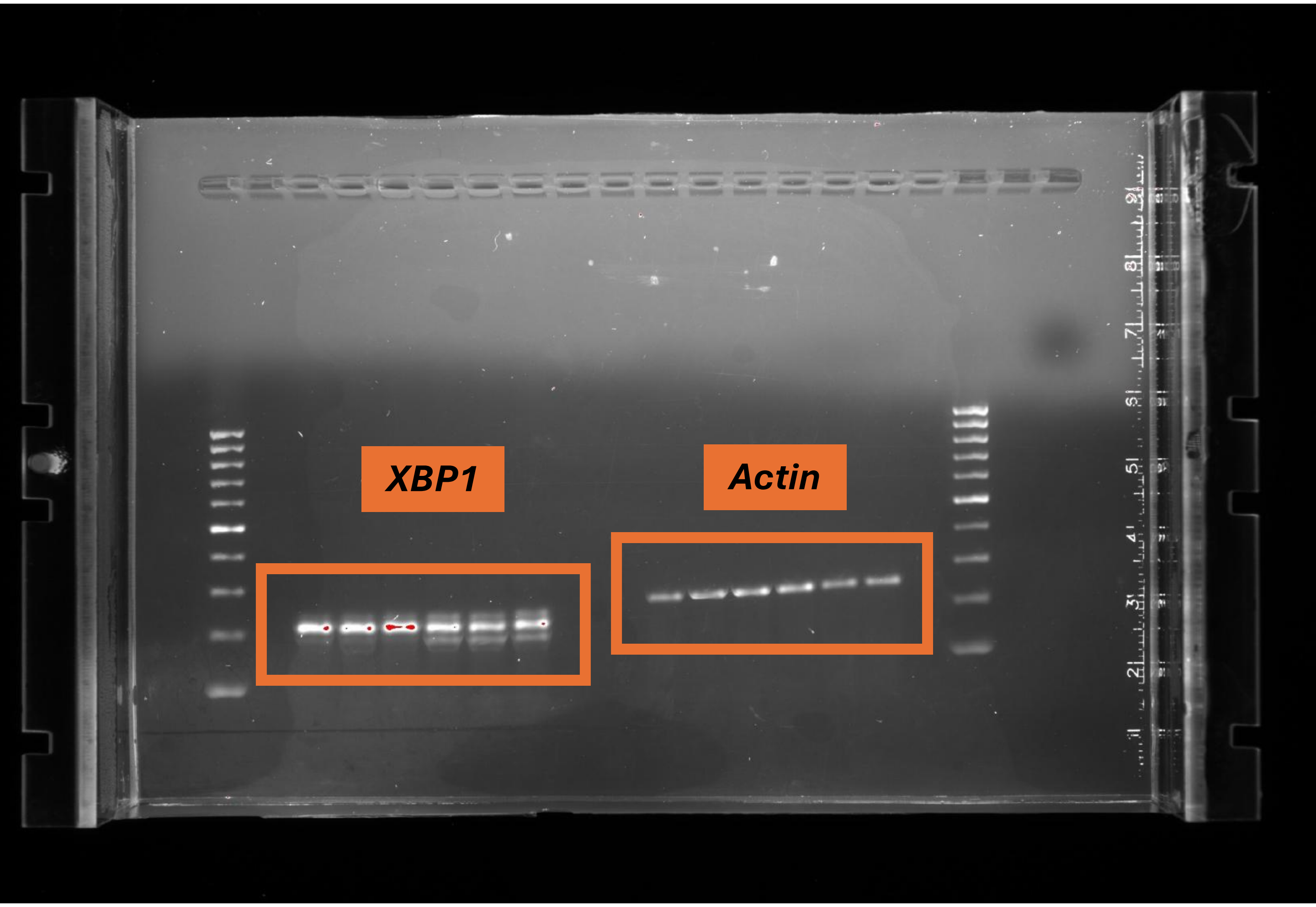

Full unedited gel for Supplemental Figure 6D

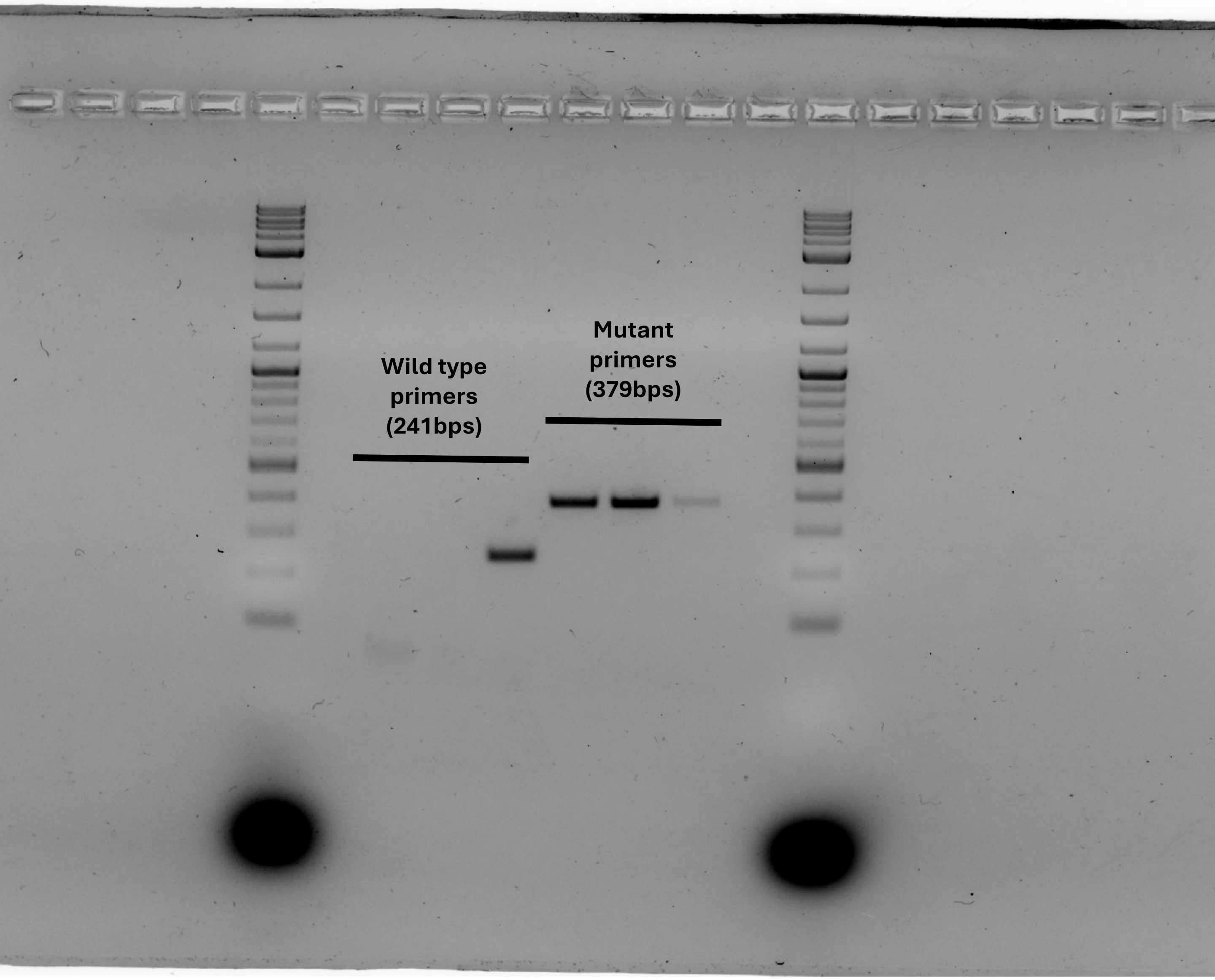

Supplement: Unedited blot and gel images [file jci-135-191074-s054.pdf]
